# ESWT Diminishes Axonal Regeneration following Repair of the Rat Median Nerve with Muscle-In-Vein Conduits but Not after Autologous Nerve Grafting

**DOI:** 10.3390/biomedicines10081777

**Published:** 2022-07-22

**Authors:** Johannes C. Heinzel, Viola Oberhauser, Claudia Keibl, Barbara Schädl, Nicole V. Swiadek, Gregor Längle, Helen Frick, Cyrill Slezak, Cosima Prahm, Johannes Grillari, Jonas Kolbenschlag, David Hercher

**Affiliations:** 1 Department of Hand-, Plastic, Reconstructive and Burn Surgery, BG Klinik Tuebingen, University of Tuebingen, Schnarrenbergstraße 95, 72076 Tuebingen, Germany; jheinzel@bgu-tuebingen.de (J.C.H.); cprahm@bgu-tuebingen.de (C.P.); jkolbenschlag@bgu-tuebingen.de (J.K.); 2 Ludwig Boltzmann Institute for Traumatology, The Research Center in Cooperation with AUVA, Donaueschingenstraße 13, 1200 Vienna, Austria; viola.oberhauser@trauma.lbg.ac.at (V.O.); claudia.keibl@meduniwien.ac.at (C.K.); barbara.schaedl@trauma.lbg.ac.at (B.S.); nicole.swiadek@trauma.lbg.ac.at (N.V.S.); gregor.laengle@gmx.at (G.L.); helen.frick@yahoo.de (H.F.); CSlezak@uvu.edu (C.S.); johannes.grillari@trauma.lbg.ac.at (J.G.); 3 Austrian Cluster for Tissue Regeneration, 1200 Vienna, Austria; 4 Core Facility Morphology, University Clinic of Dentistry, Medical University of Vienna, 1090 Vienna, Austria; 5 Department of Physics, Utah Valley University, Orem, UT 84058, USA; 6 Institute of Molecular Biotechnology, Department of Biotechnology, BOKU—University of Natural Resources and Life Sciences, Muthgasse 18, 1190 Vienna, Austria

**Keywords:** nerve repair, median nerve, rat, autologous nerve graft, muscle-in-vein conduit, extracorporeal shockwave therapy, grasping test, gait analysis, CatWalk, nerve regeneration

## Abstract

Investigations reporting positive effects of extracorporeal shockwave therapy (ESWT) on nerve regeneration are limited to the rat sciatic nerve model. The effects of ESWT on muscle-in-vein conduits (MVCs) have also not been investigated yet. This study aimed to evaluate the effects of ESWT after repair of the rat median nerve with either autografts (ANGs) or MVCs. In male Lewis rats, a 7 mm segment of the right median nerve was reconstructed either with an ANG or an MVC. For each reconstructive technique, one group of animals received one application of ESWT while the other rats served as controls. The animals were observed for 12 weeks, and nerve regeneration was assessed using computerized gait analysis, the grasping test, electrophysiological evaluations and histological quantification of axons, blood vessels and lymphatic vasculature. Here, we provide for the first time a comprehensive analysis of ESWT effects on nerve regeneration in a rat model of median nerve injury. Furthermore, this study is among the first reporting the quantification of lymphatic vessels following peripheral nerve injury and reconstruction in vivo. While we found no significant direct positive effects of ESWT on peripheral nerve regeneration, results following nerve repair with MVCs were significantly inferior to those after ANG repair.

## 1. Introduction

Peripheral nerve injuries implicate severe physical [1,2,3,4] and psychosocial impairments [5,6] for the affected patients. Depending on the degree of nerve injury, surgical treatment may be necessary to restore the affected nerve’s function, but if nerve continuity has been lost entirely, e.g., through neurotmesis, a surgical intervention is obligatory [7,8]. Reconstruction of segmental nerve injuries poses another clinical problem, given the influence of graft length and scarring at the coaptation sites on nerve regeneration [9,10,11]. While nerve autografts (ANGs) are considered the gold-standard treatment option for segmental nerve injuries, their use is restricted by their limited availability throughout the body, donor site morbidity resulting from harvesting them and specific requirements in regard to graft diameter and vascularization [12,13]. Therefore, noninvasive treatment options to enhance axonal regeneration, target organ reinnervation and functional recovery [14] are sought for by the scientific community [15,16]. Among these approaches, extracorporeal shockwave therapy (ESWT) was reported by several authors to exert significant pro-regenerative effects on lesioned peripheral nerves [17,18,19,20]. Shockwaves, sonic pulses with high energy impact, exert their effects on target tissues by biochemical changes induced by mechanotransduction. Among these effects, improved vascularization via activation of nitric oxide synthase (NOS), increased expression of growth factors like activating transcription factor 3 (ATF-3) and growth-associated phosphoprotein 43 (GAP-43), local anti-inflammatory effects and influencing of target cells, too, are thought to be the main drivers for improved tissue and nerve regeneration following ESWT [21,22,23]. Schwann cells proliferation and phenotype is also directly influenced by ESWT, enhancing peripheral nerve regeneration through activation of these glial cells [18,24,25,26]. Application of ESWT to improve nerve regeneration was first described by Hausner et al. in a rat model of sciatic nerve autograft repair [27]. The sciatic nerve injury model, especially in case of neurotmesis injuries, has several drawbacks mostly related to compromised animal welfare due to the onset of neuropathic pain, joint contractures and automutilation [28,29]. As the sciatic nerve supplies innervation both to flexor and extensor muscles of the hind paw, misdirection of axons can easily occur following neurotmesis injuries in which by definition the fascicular structure is lost. Misdirected axons in turn will either innervate the wrong target organ, e.g., an efferent axon regrowing into the skin or a muscle acting antagonistic to the axon’s original target organ. In consequence, the subtle balance of agonistic and antagonistic muscles will be lost, severely impairing functional recovery following nerve injury [30,31]. In conclusion the overall potential of functional recovery is limited in rats with sciatic nerve injury in addition to difficulties evaluating it due to the aforementioned reasons [32,33]. The median nerve model of the rat which was first described by Bertelli [34,35] about 30 years ago offers a valid alternative given that the occurrence of limb contractures, severe neuropathic pain and automutilation is far less frequently observed in comparison to sciatic nerve injuries [36]. Furthermore, functional recovery can be evaluated by means of the grasping test [37], staircase test [38] and computerized gait analysis [39] in addition to electrophysiological testing and histological analysis of the regenerating nerve [40,41]. Besides these considerations regarding the choice of an appropriate animal model, the use of ESWT in preclinical studies of segmental nerve injuries remains limited to ANGs, and the effects of ESWT on non-nervous grafts have not been reported yet. Muscle-in-vein conduits (MVCs) which were first described by Bertelli in the 1990s are an alternative to reconstruct segmental nerve injuries and promising results have been published following their clinical application [42,43,44]. We have recently reviewed the results of nerve repair by means of MVCs both in preclinical and clinical research [45]. This review’s main findings were significant differences in regard to functional recovery between animal studies and human studies utilizing MVCs to reconstruct segmental lesions of peripheral nerves. We hypothesized different experimental settings and profound inter-species differences in neurobiology to be the main reasons for this observation. In conclusion, we advised for further studies to investigate the results of nerve reconstruction by means of MVCs and potential approaches to tackle the likely biological hurdles impeding nerve regeneration through them. Given this lack of studies investigating potential pro-regenerative effects of ESWT in a murine model of forelimb nerve injury on the one hand and the interplay of ESWT and nerve reconstruction by means of MVCs on the other, we designed an in vivo study addressing both these research questions at hand. It was the aim of our study to test the hypothesis that a single postoperative application of ESWT can immediately enhance peripheral nerve regeneration following reconstruction of the rat median nerve with either ANGs or MVCs.

## 2. Materials and Methods

### 2.1. Animals and Surgery

The experimental protocol was approved beforehand, on 23 July 2019, by the Animal Protocol Review Board of the City Government of Vienna (Magistrate’s office No. 58, project identification code MA58-421715-2019-16). All the procedures were carried out in full accord with the Helsinki Declaration on Animal Rights and the Guide for the Care and Use of Laboratory Animals of the National Institutes of Health.

Fifty-six male Lewis rats (Janvier Labs, Le Genest-Saint-Isle, France) weighing 280–350 g were kept in groups of two or three in appropriate cages according to the internal standard operating procedures. The animals had access to food and water ad libitum. After the rats were allowed to get accustomed to their new surroundings for 7 days prior to any experimental handling and after completing a 7-day training period on the CW device, they were randomly assigned to the following groups: median nerve reconstruction with autologous nerve grafts (ANGs) (total *n* = 29) and median nerve reconstruction with MVCs (total *n* = 27). Both groups were further subdivided into a group of animals which received ESWT, ANG + ESWT (*n* = 15) and MVC + ESWT (*n* = 11), a group of animals which received no additional treatment and control groups that received an ANG (*n* = 14) or an MVC (*n* = 16). Therefore, a total of four groups of animals were investigated in this study.

After random group allocation, the rats underwent bilateral surgery of the median nerve under an operation microscope (Leica M651, Leica Microsystems, Vienna, Austria). A 7 mm segment of the left and right nerves each was removed by performing a transection about 1.5 mm proximal to the position where it is crossed over by the brachial artery and vein and another transection 7 mm proximal to the first one. On the right side, the gap was bridged with either the original nerve segment in reverse fashion as a homotopic ANG or an MVC. MVCs were prepared by introducing several muscle fibers of the left gracilis muscle into a segment of the epigastric vein as described elsewhere [38]. ANGs and MVCs were coaptated with the proximal and distal stump of the median nerve with two sutures per coaptation site (Ethilon, 10-0, Ethicon-Johnson & Johnson, Brussels, Belgium). On the left side, the nerve defect remained unreconstructed to serve as an internal control group. To prevent spontaneous regeneration, the distal nerve stump was sutured into the short head of the biceps muscle. The postoperative observation period lasted 12 weeks. At the end of the postoperative observation periods, the rats were sacrificed in deep anesthesia induced as described above via intracardial puncture and administration of an overdose of sodium thiopental.

### 2.2. Application of ESWT

Following median nerve reconstruction with an ANG or an MVC, the rats in both the ANG + ESWT group (*n* = 15) and the MVC + ESWT group (*n* = 11) received 300 impulses (3 Hz, 0.1 mJ/mm^2^) of ESWT (OP 155 connected to Orthogold 100, MTS Medical, Konstanz, Germany) while still in deep anesthesia to prevent movement-induced artifacts. Focused application of ESWT was facilitated by the use of a 3D-printed customized device in which the right forelimb was introduced and fixed using a noose made from an elastomer (Supplementary Figure S1). The area between the applicator and the rat’s right forelimb was filled with an ultrasonic transmission gel to guarantee adequate and reproducible transmission of impulses.

### 2.3. Functional Analysis

#### 2.3.1. Reflex-Based Grasping

Motor function of the superficialis finger flexor muscle (FDS) and the deep finger flexor muscle (FDP) was evaluated weekly by means of the grasping test as originally described by Bertelli [46] and modified by us [39] and other authors [38]. As the FDS and the FDP in rats are predominantly innervated by the median nerve, the ability to flex the toes of the forelimbs is mediated by this nerve [39,41,46]. We recorded three trials per week, and only those trials were deemed valid in which no flexion of the elbow (biceps muscle) or wrist (flexor carpi ulnaris muscle and flexor carpi radialis muscle) were evident. Return of the grasping ability in general was graded as described by Stößel et al.: 1/3: no observable toe flexion, 2/3: toe flexion without measurable strength when forced to pull the bar, 3/3: toe flexion with measurable strength when forced to pull the bar [38].

#### 2.3.2. CatWalk XT Gait Analysis

To evaluate changes in gait behavior, computerized gait analysis was performed biweekly using the CatWalk XT gait analysis system as described elsewhere [39,47,48]. The device consists of a walkway with a glass floor which is illuminated by green and red ceiling light sources. While the green light source illuminates a crossing rat’s or mice’s paws, the red ceiling light’s provides contrast for the animal’s body contour. The acquired images are then recorded by a fully automated camera mounted underneath the glass plate and processed by the system’s software. During the course of the seven-day training period, the animals were habituated to cross the walkway at a speed between 50 and 100 cm/s [39]. Following completion of each data acquisition session, the animals were rewarded with 1–2 pellets of cereals. We assessed the following parameters: print area ratio of the right front paw (RF) and the right hind paw (RH) (%), print length ratio RF/RH (%), print width ratio RF/RH (%), swing speed ratio (RF/RH) (%), swing time ratio RF/RH (%), duty cycle ratio RF/RH (%), stand index ratio RF/RH (%), front paw base of support (FP BoS) (%) and ulnar abduction of the RF [39].

#### 2.3.3. Electrophysiological Analysis

At the end of the twelve-week observation period, the rats underwent electrophysiological evaluations with a Neuromax EMG device (Natus, Middleton, WI, US) as described elsewhere [39]. Briefly, the right median nerve was gently freed from its surrounding tissue. The recording electrode was placed inside the flexor digitorum superficialis muscle while the reference electrode was placed in the ipsilateral paw. The grounding electrode was subcutaneously inserted in the right hind limb. Using a micromanipulator, a bipolar stimulation electrode was positioned 2–3 mm proximal to the proximal coaptation site. Latency and compound muscle action potential (CMAP) of the flexor digitorum superficialis muscle were measured using supramaximal stimulation. The measurements were normalized with the animal’s core temperature which was assessed rectally.

### 2.4. Wet Muscle Weight

Following sacrifice of the animals, both right and left flexor digitorum superficialis muscles were harvested and weighted. The weight of the right FDS muscle was normalized both to the weight of the contralateral, chronically denervated muscle as well as to the animal’s body weight.

### 2.5. Histological Analysis

To obtain the correct position as well as distal and proximal orientation of the nerves, they were pinned with minutius needles on small Styropor stubs. For histochemical and immunohistochemical staining, the nerves were fixed in 4% buffered formalin for 24 h at room temperature and afterwards rinsed in tap water for 1 h. Dehydration with an uprising ethanol series was performed, beginning with 50% EtOH for 1 h, followed by 70% EtOH. Then, the samples were transferred to a vacuum infiltration processor (Sakura, TissueTek ^®^ VIP) and, after further dehydration of the samples, infiltrated with paraffin via the intermedium of xylene. The nerve samples were cut in 4 µm thin cross-sections with an Microm HM355S (Thermo Scientific, Waltham, MA, US). After drying the sections overnight in a 37 °C oven, the slides were deparaffinized and rehydrated for staining with different methods. The nuclei were stained in grey using Weigert’s iron hematoxylin. After staining, the sections were dehydrated and permanently embedded with Shandon Consul-Mount (Thermo Scientific). Starting immunohistochemical staining, the sections were first pretreated with different antigen retrieval protocols. For S100 (Agilent, Santa Clara, CA, US, Z0311), the sections were incubated with Pepsin (Sigma-Aldrich, St. Louis, MO, US) for 10 min at 37 °C in a humidified chamber. The sections for podoplanin (Relia Tech, Wolfenbüttel, NI, Germany, 104-M40) staining were steamed in a pH 6 sodium citrate buffer (0.1 M) for 20 min, for CD31 (Thermo Scientific, Waltham, MA, US, PA5-16301)—in EDTA buffer (0.1 M) at pH 9. After antigen retrieval, the sections were blocked using Bloxall^®^ (Vector Labs, Newark, CA, US) for 10 min. Then, the primary antibodies were applied for 1 h at room temperature (S100, 1:1600; podoplanin, 1:2000; CD31 1:50), which was followed by incubation of the secondary antibodies for 30 min at room temperature using an HRP-conjugated anti-mouse system (Immuno Logic, VWRKDPVM110HRP) for S100 and podoplanin. For CD31, an anti-rabbit HRP-conjugated antibody was used (Immuno Logic, VWRKDPVR110HRP). Detection of the staining was performed with ImmPACTTM NovaREDTM (Vector Labs, Newark, CA, US). Then, the sections were counterstained with hematoxylin and, after dehydration, permanently embedded with Shandon Consul-Mount (Thermo Scientific, Waltham, MA, US).

#### Automated Quantification of Axons, Lymphatic and Blood Vessels

We employed automated deep learning-based image analysis to quantify the axon and lymphatic vessel counts in whole-slide scans of histological cross-sections. The IKOSA platform (KML Vision) was adopted to train two state-of-the-art deep neural network models in a supervised fashion. To quantify the lymphatic vessels, we applied our previously trained model to our image data as described elsewhere [49].

A second model was trained to segment axons in digital images. To improve the ground truth data annotation quality, regions of interest (ROIs) were defined to restrict the area where axons were marked using the annotation tools provided by IKOSA. A set of 54 whole-slide scans containing 149 ROIs was randomly split into training (48 images, 116 ROIs) and validation (six images, 33 ROIs) data. The model training converged after 4 h 43 min on GPU infrastructure. The validation performance at the axon instance count was 95.4% recall and 94.2% precision. See Supplementary Table S1 and Supplementary Figure S2 for more details on the dataset and validation statistics.

Blood vessel counts in the cross-sections were reported as manual counts.

### 2.6. Statistical Analysis

All the statistical analyses were performed using IBM SPSS Version 26 (International Business Machines Corporation, Armonk, NY, USA). For each parameter, normal distribution was tested by means of the Kolmogorov–Smirnov test. Homogeneity of variances was tested with Levene’s test. In case both criteria were met, the data were compared with parametric tests, e.g., one-way analysis of variance (ANOVA). This was followed by Tukey’s post hoc test. Otherwise, nonparametric comparisons, e.g., the Kruskal–Wallis test, followed by the Dunn–Bonferroni post hoc test were used for comparison for more than two groups. Sub-analysis of the groups (ANG vs. MVI; ANG vs. ANG + ESWT, MVI vs. MVI + ESWT) was performed with the Mann–Whitney U test in case of nonnormally distributed data; otherwise, Student’s t-test was used. Repeated measures of the same sample were compared with the nonparametric Friedman test. *P*-values < 0.05 were considered statistically significant. All the values were expressed as the means ± one standard error of the mean (SEM).

## 3. Results

### 3.1. Reflex-Based Grasping

By using the grasping test, we aimed to evaluate the return of the general grasping ability in general and of grasping strength in particular as both depend on reinnervation of the flexor digitorum superficialis muscle (FDS), one of the median nerve’s target organs.

One animal had to be excluded from statistical analysis because the preoperatively recorded data were lost due to a technical error. Animal motivation to participate in the procedure showed some substantial fluctuations over time in our study. Of all the rats (*n* = 55), 34 animals showed no motivation to participate in the grasping test at least once over the entire observation period. In the ANG and MCV groups, 11 animals in each case were reluctant to grasp the bar at least once. The same applied to eight animals in the ANT+ group and four animals in the MVC+ group, respectively. Therefore, these animals had to be excluded from statistical analysis.

**Figure 1:**
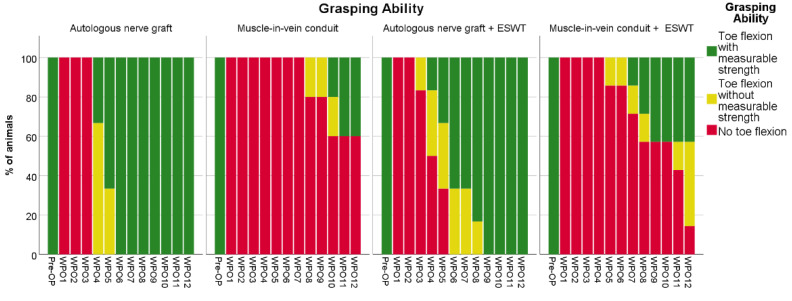
Recovery of the grasping ability following reconstruction of the right median nerve during the postoperative 12-week observation period. Autologous nerve graft (*n* = 3); muscle-in-vein conduit (*n* = 5); autologous nerve graft + ESWT: autologous nerve graft with postoperative extracorporeal shockwave therapy (*n* = 6); muscle-in-vein conduit + ESWT: muscle-in-vein conduit with postoperative extracorporeal shockwave therapy (*n* = 7). WPO: postoperative week.

Comparison of the remaining 21 animals (Figure 1) of the ANT (*n* = 3), MVC (*n* = 5), ANT+ (*n* = 6) and MVC+ (*n* = 7) groups revealed significant differences regarding the overall grasping ability during the course of the observational period (Table 1). While no animal had displayed toe flexion until WPO2 in any group, functional recovery was observed to occur fastest in the two groups of animals which underwent median nerve reconstruction with an ANG. Starting from WPO6, all the animals in the ANG group regained the ability to grasp the bar with measurable force.

In the ANG + ESWT group, five out of the six animals regained full motor function, i.e., a grasping rating of 3/3, in WPO8, with the remaining animal displaying this ability starting from WPO9.

The animals which had undergone nerve reconstruction with an MVC without additional ESWT showed no sign of functional recovery until WPO8. By the end of the observation period, two out of the five animals had regained grasping ability. While both these animals were able to flex their toes with measurable force, the remaining three animals did not show any sign of functional recovery regarding voluntary grasping ability.

In the MVC + ESWT group, the first signs of motor recovery became apparent in WPO5, with one animal regaining the ability to grasp the bar without measurable strength. In WPO12, six of the seven animals displayed signs of functional recovery, i.e., a grasping rating of 2/3 or 3/3.

**Figure 2:**
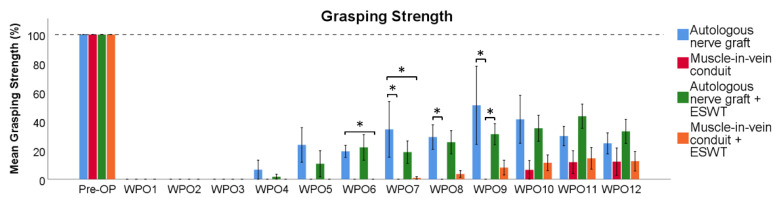
Recovery of grasping strength following reconstruction of the right median nerve. Statistical analysis was performed with the nonparametric Kruskal–Wallis test and the Dunn–Bonferroni post hoc test. Autologous nerve graft (*n* = 3); muscle-in-vein conduit (*n* = 5); autologous nerve graft with postoperative extracorporeal shockwave therapy (*n* = 6); muscle-in-vein conduit with postoperative extracorporeal shockwave therapy (*n* = 7). WPO: postoperative week; * *p* < 0.05.

Regarding the evaluation of the mean grasping strength as compared to baseline recordings (Figure 2), no grasping strength was recordable until WPO4 in any group. In WPO4 and WPO5, the grasping strengths measured in the ANG group and the ANG + ESWT group were not statistically significantly different from each other, while there was still no grasping strength measurable in both the MVC and MVC + ESWT group. Six weeks postoperatively, the animals of the ANG group recovered significantly (*p* < 0.05) more grasping strength than those of the MVC + ESWT group. This difference was not statistically significant (*p* = 0.075) when comparing the ANG group to the MVC group. The difference between the mean grasping strength ratios of the ANG + ESWT group and the MVC + ESWT group was also not statistically significant (*p* = 0.058). In WPO7, the rats in the ANG group recovered a significantly (*p* < 0.05) greater grasping strength than the rats in both the MVC group and the MVC + ESWT group, respectively. Eight weeks postoperatively, the mean grasping strength in the ANG + ESWT group was significantly (*p* < 0.05) higher compared to the MVC group. In WPO9, the animals in the MVC group had still not recovered any grasping strength which was statistically significant (*p* < 0.05) compared to the two groups which underwent median nerve repair with an ANG or an ANG + ESWT, respectively. Ten weeks after median nerve reconstruction, the rats in all the groups recovered measurable grasping strength, but there were no statistically significant differences observable between the groups until the end of the observation period.

### 3.2. CatWalk XT Gait Analysis

Computerized gait analysis was used in this study in order to evaluate recovery of the sensory and motor function following median nerve resection and immediate reconstruction.

#### 3.2.1. Print Area 

**Figure 3:**
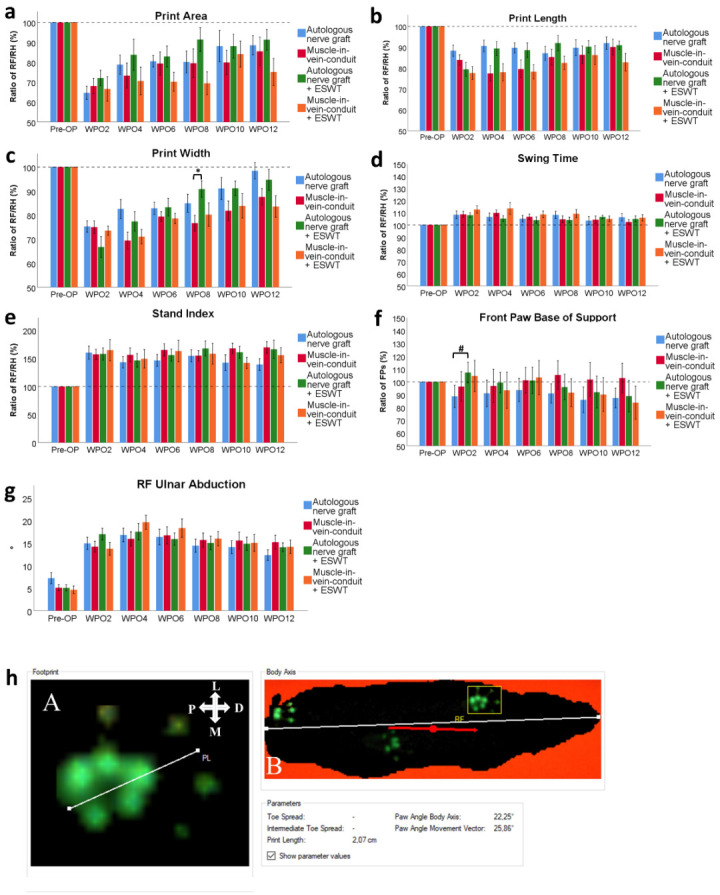
Results of computerized gait analysis via CatWalk XT version 10.6 following resection of a 7 mm segment of the right median nerve and immediate reconstruction with an autologous nerve graft (*n* = 13), a muscle-in-vein conduit (*n* = 16), an autologous nerve graft + ESWT (autologous nerve graft with postoperative extracorporeal shockwave therapy) (*n* = 15) or a muscle-in-vein conduit + ESWT (muscle-in-vein conduit with postoperative extracorporeal shockwave therapy) (*n* = 11). The assessed parameters included the paw print area of the right front paw (**a**), the print length ratio of the right front paw (**b**), the print width ratio of the right front paw (**c**), the swing time ratio of the right front paw (**d**), the stand index ratio of the right front paw (**e**), the front paw base of support ratio (**f**) and ulnar abduction of the right front paw (**g**) with a schematic of how to assess this latter parameter (**h**): Evaluation of the degree of external paw rotation by measuring the Paw Angle Body Axis. This is the angle between the orientation of the front paw (**A**) and the animal’s body axis (**B**). The white line in panel **A** defines the orientation of the paw. The white line in panel **B** defines the animal’s body axis. The red line defines the animal’s body movement vector, which is calculated by linear regression of the animal’s center of gravity in the recorded frame and in the three frames preceding it. The white cross indicates the orientation of the respective paw in relation to the rat’s body axis; P, proximal; D, distal; M, medial; L, lateral. [39]). Statistical analysis was performed with the nonparametric Kruskal–Wallis test and the Dunn–Bonferroni post hoc test. For subgroup analysis of identical reconstructive approaches, the Mann–Whitney U test was used. FPs: front paws, * *p* < 0.05, # *p* < 0.05 in subgroup analysis only.

There were no significant differences between the four groups regarding the print area ratio RF/RH (Figure 3a) during the entire course of the 12-week observation period. There was, however, a trend towards better functional recovery in two groups in which the median nerve was reconstructed with an ANG. The animals which received an MVC and additional ESWT showed a trend towards a lower print area ratio RF/RH.

#### 3.2.2. Print Length 

Print length ratio RF/RH (Figure 3b) was not significantly different between the groups over the entire course of the observational period. In accordance with the course of the print area ratio, a trend towards better functional recovery was apparent in the ANG and ANG + ESWT groups, especially at WPO4 and WPO6, respectively.

#### 3.2.3. Print Width 

Print width ratio RF/RH (Figure 3c) was markedly decreased in all the groups following segmental median nerve injury. Starting from WPO4, there was a trend towards a higher print width ratio RF/RH in the two groups which underwent median nerve reconstruction with an ANG compared to the groups in which an MVC was used. At WPO8, the rats which underwent median nerve reconstruction with an ANG and received additional ESWT had a significantly (*p* < 0.05) higher print width ratio than those which underwent median nerve reconstruction with an MVC but without additional ESWT.

#### 3.2.4. Swing Speed (Data Not Shown)

Analysis of the swing speed ratio RF/RH did not reveal any marked alterations of this parameter (statistical differences) between the groups compared to preoperative measurement in any group.

#### 3.2.5. Swing Time 

In regard to the swing time ratio RF/RH (Figure 3d), no statistically significant differences were observable between the four groups at any timepoint. The parameter was increased in all the groups following right median nerve injury and reconstruction.

#### 3.2.6. Duty Cycle (Data Not Shown)

There were no significant differences detectable between the groups in regard to the duty cycle ratio RF/RH (data not shown) over the entire course of the observation period.

#### 3.2.7. Stand Index 

Stand index ratio RF/RH (Figure 3e) was markedly increased following median nerve resection and immediate reconstruction in all the groups. Statistical analysis revealed no significant differences between groups.

#### 3.2.8. FP BoS 

Differences in the FP BoS (Figure 3f) at WPO2 nearly reached statistical significance (*p* = 0.061) between the ANG-treated animals and the animals of the ANG + ESWT group when comparing all the groups. In the subgroup analysis of identical reconstructive techniques, this difference was highly statistically significant (*p* < 0.05).

#### 3.2.9. Ulnar Abduction of the Right Front Paw 

Ulnar abduction of the right front paw (Figure 3g), measured as published previously by our group (Figure 3h) [39], was not statistically significantly different between the groups at any pre- or postoperative timepoint.

### 3.3. Appearance of the Reconstructed Median Nerve at WPO12

**Figure 4:**
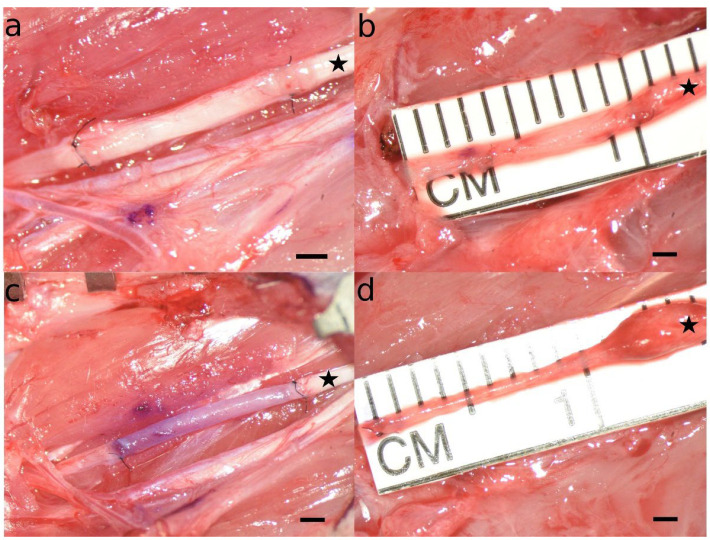
Microscopic appearance of autologous nerve grafts (**a**,**b**) and muscle-in-vein conduits (**c**,**d**) immediately after nerve reconstruction (**a**,**c**) and at the timepoint of sacrifice twelve weeks after the initial surgery (**b**,**d**). Note that while the autologous neve grafts at WPO12 were comparable both in length and diameter to the originals grafts sutured to the stumps of the median nerve during the initial surgery, the muscle-in-vein-conduits appeared markedly thinner and stretched at the timepoint of sacrifice as compared to the initial surgery. Additionally, a prominent coaptation neuroma was observable at the proximal coaptation site in almost all the cases a muscle-in-vein conduit was used for nerve reconstruction. The proximal part of the reconstructed nerve is marked with an asterisk. Scale bar = 1 mm.

During the initial surgery, we took images of the ANG or MVC we used to reconstruct the right median nerve (Figure 4a,c). When the rats were sacrificed in deep anesthesia twelve weeks postoperatively, the reconstructed right median nerve was inspected microscopically to assess the appearance of the regenerated tissue (Figure 4b,d). These differences were also compared to the images taken during the initial surgery. While there were no gross differences observable between identical reconstructive techniques, e.g., ANG vs. ANG + ESWT and MVC vs. MVC + ESWT, respectively, we identified some major distinctive features between the animals which underwent median nerve reconstruction with an ANG (Figure 4a,b) or an MVC (Figure 4c,d). While the ANGs at WPO12 were comparable in diameter and length to the grafts initially sutured between the nerve stumps, the MVCs appeared significantly stretched, in some cases reaching lengths of more than 10 mm. Additionally, we noticed prominent neuroma formation at the proximal coaptation site in almost all the cases. The MVCs were also markedly narrower in their distal segments when compared to the proximal nerve segment. This trend was also observable as the more distal parts of the MVC were inspected, reaching the smallest diameters at the site of distal nerve coaptation.

### 3.4. Electrophysiological Evaluations

**Figure 5:**
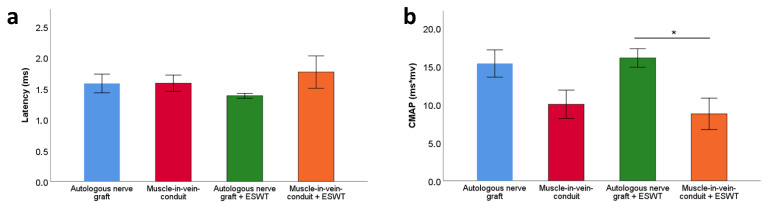
Distal motor latency (**a**) and compound muscle action potential area (**b**) of the reconstructed right median nerve 12 weeks following resection of a 7 mm segment of the right median nerve and immediate reconstruction with an autologous nerve graft (*n* = 14), a muscle-in-vein conduit (*n* = 11), an autologous nerve graft + ESWT (autologous nerve graft with postoperative extracorporeal shockwave therapy) (*n* = 15) or a muscle-in-vein conduit + ESWT (muscle-in-vein conduit with postoperative extracorporeal shockwave therapy) (*n* = 8). Results of the electrophysiological evaluations were compared between the groups with the nonparametric Kruskal–Wallis test and the Dunn–Bonferroni post hoc test. CMAP: compound muscle action potential; * *p* < 0.05.

Statistical comparison of distal motor latencies (Figure 5a) and CMAP areas (Figure 5b) at WPO12 revealed no statistically significant differences between the groups in regard to distal motor latency. Regarding the CMAP area, there was a trend towards higher values observable in the two groups which underwent median nerve reconstruction with an ANG. The values were significantly (*p* < 0.05) higher in the ANG + ESWT group when compared to the MVC + ESWT group. The CMAP values in the ANG group and the MVC group ranged between those two, but there were no observable statistically significant differences.

### 3.5. Wet Muscle Weight

**Figure 6:**
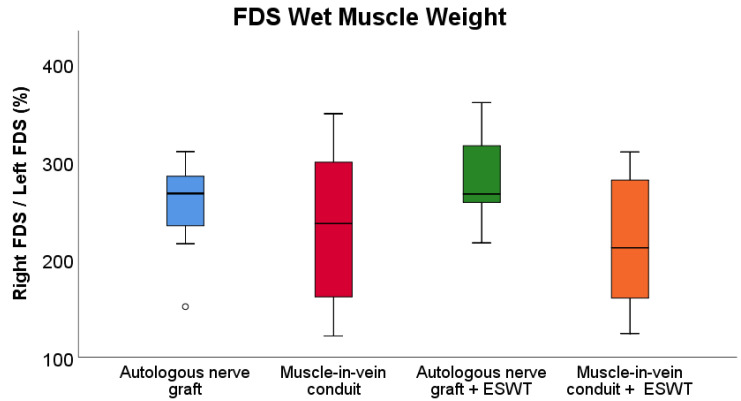
Comparison of the wet muscle weight ratio of the right and left flexor digitorum superficialis muscles at postoperative week 12 following bilateral resection and unilateral reconstruction of the right median nerve. Autologous nerve graft: *n* = 14; muscle-in-vein-conduit: *n* = 15, autologous nerve graft + ESWT: autologous nerve graft with postoperative extracorporeal shockwave therapy (*n* = 15); muscle-in-vein-conduit + ESWT: muscle-in-vein conduit with postoperative extracorporeal shockwave therapy (*n* = 11). Statistical analysis was performed with the nonparametric Kruskal–Wallis test and the Dunn–Bonferroni post hoc test. For subgroup analysis of identical reconstructive approaches, the Mann–Whitney U test was used. FDS: flexor digitorum superficialis muscle.

Despite a trend of higher FDS muscle weight ratios (Figure 6) in both the ANG group and ANG + ESWT group, respectively, there were no statistically significant differences (*p* = 0.05) observable between the four groups. The FDS muscle weight ratios in the MVC and MVC + ESWT group were lower than in the ANT group and the ANT + ESWT group with a trend for the lowest values in the groups of rats which received an MVC + ESWT.

### 3.6. Histological Evaluations

**Figure 7:**
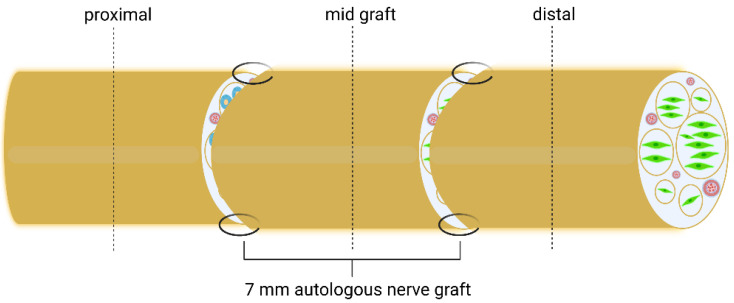
Schematic of the repaired median nerve with histological sections and their respective localization indicated as proximal, start graft, mid graft and distal. Dotted lines indicate histological cutting planes [49].

The schematic indicating the histological sections of the reconstructed median nerve taken at WPO12 is depicted in Figure 7.

#### 3.6.1. Number of Axons

**Figure 8:**
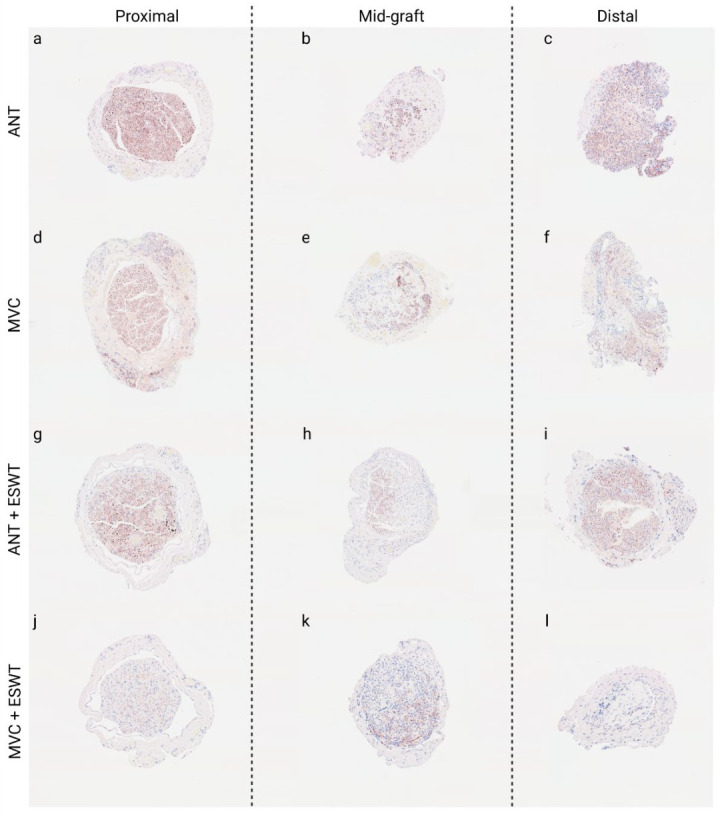
Representative photomicrographs of anti-neurofilament-stained consecutive cross-sections through the regenerated tissue in the proximal nerve segment (**a**,**d**,**g**,**j**), the middle of the nerve graft (**b**,**e**,**h**,**k**) and the distal nerve graft (**c**,**f**,**i**,**l**) at 12 weeks post-surgery. Autologous nerve graft (**a**–**c**); muscle-in-vein-conduit (**d**–**f**); autologous nerve graft + extracorporeal shockwave therapy (**g**–**i**); muscle-in-vein-conduit + extracorporeal shockwave therapy (**j**–**l**). Scale bar = 200 μm.

**Figure 9:**
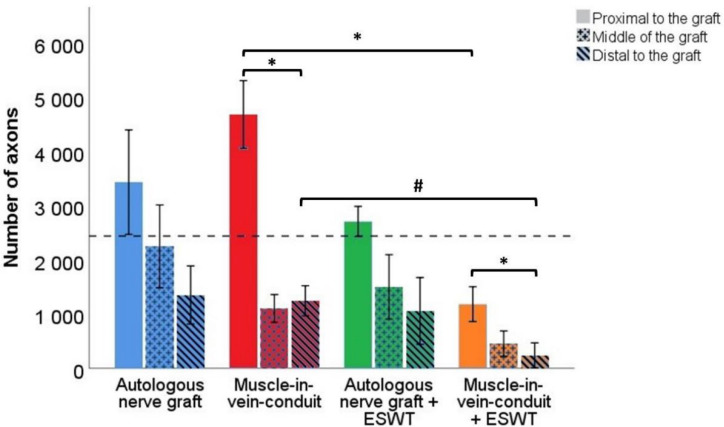
Number of axons in different segments of the reconstructed right median nerve 12 weeks following resection of a 7 mm segment of the right median nerve and immediate reconstruction with an autologous nerve graft (*n* = 5), a muscle-in-vein conduit (*n* = 6), an autologous nerve graft + ESWT (autologous nerve graft with postoperative extracorporeal shockwave therapy) (*n* = 4) or a muscle-in-vein conduit + ESWT (muscle-in-vein conduit with postoperative extracorporeal shockwave therapy) (*n* = 4). The dashed line indicates the number of axons in the uninjured median nerve at the mid-humerus level (2442 ± 19). Statistical evaluation was performed with the nonparametric Kruskal–Wallis test and the Dunn–Bonferroni post hoc test. For subgroup analysis of identical reconstructive approaches, the Mann–Whitney U test was used. Repeated measurements of the same group were compared by means of the nonparametric Friedman test. Note: * *p* < 0.05, # *p* < 0.05 in subgroup analysis only.

Representative photomicrographs of anti-neurofilament-stained cross-sections are shown in Figure 8**.** Statistical comparison of proximal nerve segments (Figure 9) revealed statistically significantly (*p* < 0.05) lower axon numbers in the proximal nerve segments of the MVCs which were treated with ESWT postoperatively (1184 ± 321) when compared to the MVCs which received no additional treatment (4683 ± 624). In accordance with this observation, more axons were found in the proximal nerve segments of untreated ANGs (3436 ± 963) than in the ANG + ESWT group (2710 ± 278), but this trend was not statistically significant. 

Axon numbers in the mid-graft segments of the ANG group (2251 ± 764), MVC group (1106 ± 255), ANG + ESWT group (1502 ± 595) and MVC + ESWT group (456 ± 233) were not statistically significantly different from each other.

The same applied to the axon numbers in the distal nerve segments. Axon numbers were highest in the ANG group (1354 ± 535) followed by the MCV group (1248 ± 277) and lowest in the MVC + ESWT (239 ± 230) group, with the ANG + ESWT group (1060 ± 615) in between.

Subgroup analysis of identical reconstructive approaches revealed a significantly (*p* < 0.05) lower number of axons in both the proximal as well as the distal nerve segments of the animals which underwent median nerve reconstruction with an MVC and additional ESWT. When we compared the number of axons within the same reconstructed nerve for each group, significantly (*p* < 0.05) lower numbers were found in the distal nerve segments of the MVC- and MVC + ESWT-treated animals when compared to the counts in the proximal nerve segment. No significant differences were found in the case of the other two groups.

#### 3.6.2. Number of Blood Vessels

Representative photomicrographs of anti-CD31-stained cross-sections of the reconstructed median nerve are shown in Supplementary Figure S3.

**Figure 10:**
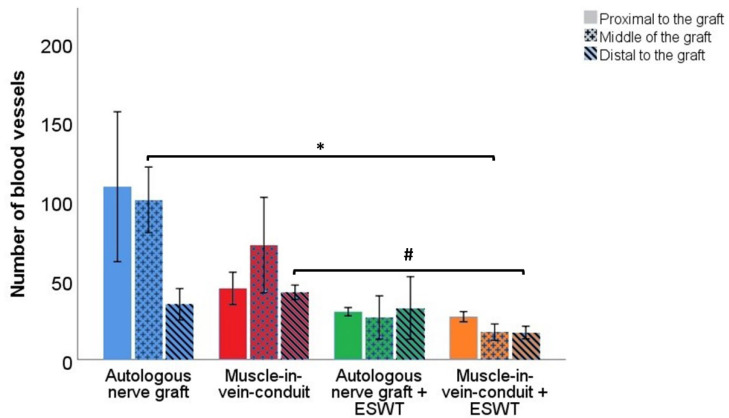
Number of blood vessels in different segments of the reconstructed right median nerve 12 weeks following resection of a 7 mm segment of the right median nerve and immediate reconstruction with an autologous nerve graft (*n* = 4), a muscle-in-vein conduit (*n* = 5), an autologous nerve graft + ESWT (autologous nerve graft with postoperative extracorporeal shockwave therapy) (*n* = 3) or a muscle-in-vein conduit + ESWT (muscle-in-vein conduit with postoperative extracorporeal shockwave therapy) (*n* = 5). Statistical evaluation was performed with the nonparametric Kruskal–Wallis test and the Dunn–Bonferroni post hoc test. For subgroup analysis of identical reconstructive approaches, the Mann–Whitney U test was used. Repeated measurements of the same group were compared by means of the nonparametric Friedman test. Note: * *p* < 0.05, # *p* < 0.05 in subgroup analysis only.

Blood vessel numbers (Figure 10) in the proximal segments of the reconstructed median nerves were not statistically significantly different between the animals of the ANG (109 ± 47), MVC (66 ± 23), ANG + ESWT (30 ± 3) or MVC + ESWT groups (27 ± 3), respectively.

The same applied to the number of blood vessels in the distal segment of the reconstructed nerve. While numbers were lowest in the MVC + ESWT group (17 ± 4), counts in the ANG (35 ± 10) group and the ANG + ESWT (33 ± 20) group were almost the same. Most blood vessels could be found in the MVC group (43 ± 5) at this level.

Regarding the number of blood vessels at the mid-graft level, significant differences were found between the animals of the ANG group (101 ± 21) and the MVC + ESWT group (17 ± 5). There were no observable statistically significant differences regarding the ANG + ESWT group (27 ± 14) or the MVC group (72 ± 30).

Subgroup analysis of identical reconstructive approaches by means of the Mann–Whitney U test revealed a statistically significantly (*p* < 0.05) lower number of blood vessels in the distal nerve segments of the MVC + ESWT-treated animals as compared to the group of animals which received an MVC without additional ESWT.

Comparison of blood vessel numbers within different nerve segments of each group revealed no statistically significant differences.

#### 3.6.3. Number of Lymphatic Vessels

**Figure 11:**
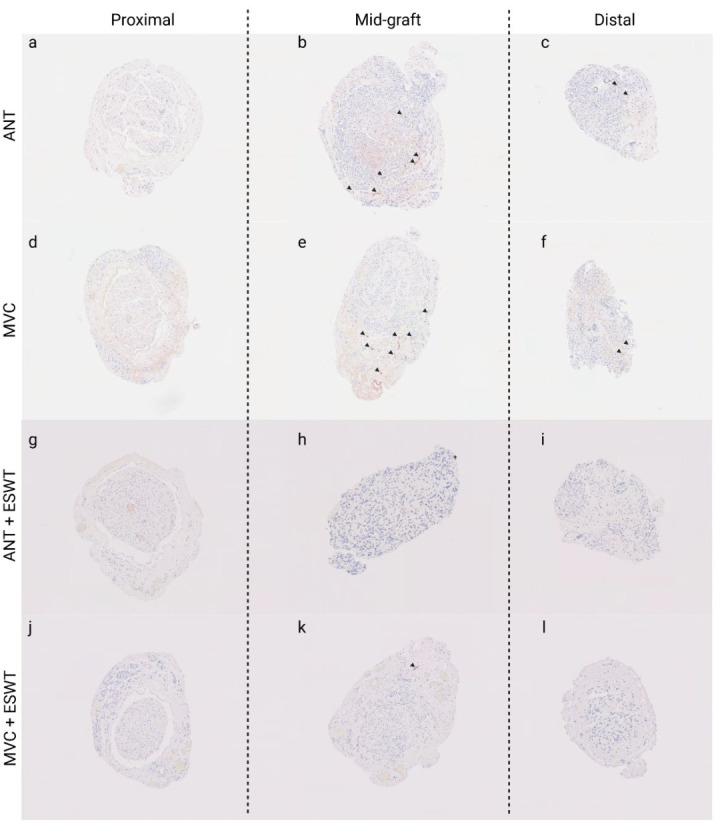
Representative photomicrographs of anti-podoplanin-stained consecutive cross-sections through the regenerated tissue in the proximal nerve segment (**a**,**d**,**g**,**j**), the middle of the nerve graft (**b**,**e**,**h**,**k**) and the distal nerve graft (**c**,**f**,**i**,**l**) at 12 weeks post-surgery. Autologous nerve graft (**a**–**c**); muscle-in-vein-conduit (**d**–**f**); autologous nerve graft + extracorporeal shockwave therapy (**g**–**i**); muscle-in-vein-conduit + extracorporeal shockwave therapy (**j**–**l**). Scale bar = 200 μm. Podoplanin-positive stained lymphatic vessels are indicated by arrowheads.

Figure 11 displays representative photomicrographs of anti-podoplanin-stained consecutive cross-sections of the reconstructed median nerve.

**Figure 12:**
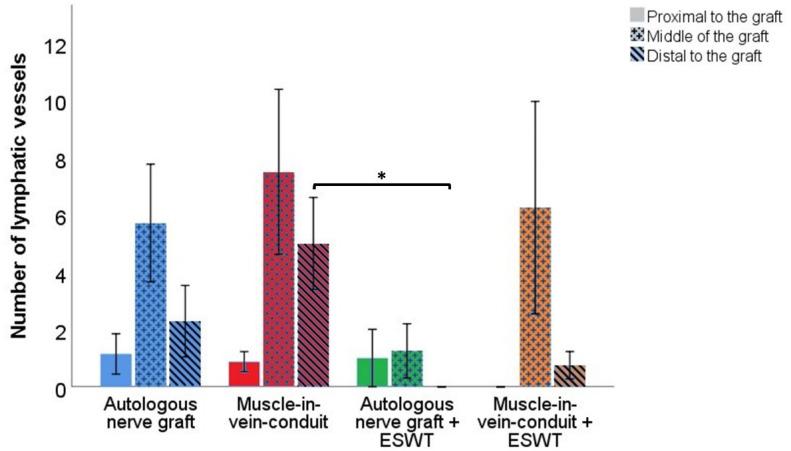
Number of lymphatic vessels in different segments of the reconstructed right median nerve 12 weeks following resection of a 7 mm segment of the right median nerve and immediate reconstruction with an autologous nerve graft (*n* = 8), a muscle-in-vein conduit (*n* = 8), an autologous nerve graft + ESWT (autologous nerve graft with postoperative extracorporeal shockwave therapy) (*n* = 5) or a muscle-in-vein conduit + ESWT (muscle-in-vein conduit with postoperative extracorporeal shockwave therapy) (*n* = 4). Statistical evaluation was performed with the nonparametric Kruskal–Wallis test and the Dunn–Bonferroni post hoc test. For subgroup analysis of identical reconstructive approaches, the Mann–Whitney U test was used. Repeated measurements of the same group were compared by means of the nonparametric Friedman test. Note: * *p* < 0.05.

Statistical comparison of the number of lymphatic vessels in different segments of the reconstructed median nerve between the groups (Figure 12) revealed no statistically significant differences between the groups in regard to the proximal and mid-graft segments.

In the proximal nerve segment, lymphatic vessel numbers in the ANG group (1.14 ± 0.70), the MVC group (0.88 ± 0.35) and the ANG + ESWT group (1.00 ± 1.00) were almost identical, whereas no lymphatic vessels could be identified within the proximal nerve segment of the MVC + ESWT-treated animals.

The numbers in the mid-graft segment were highest in the MVC group (7.50 ± 2.87), followed by the MVC + ESWT group (6.25 ± 3.70), the ANG group (5.00 ± 1.91) and the ANG + ESWT group (1.00 ± 0.77).

Statistically significant (*p* < 0.05) differences in lymphatic vessel counts could be identified in the distal nerve segments of the ANG + ESWT (0) and MVC-treated (5.00 ± 1.60) animals. There were no statistically significant differences regarding the number in the ANG group (2.00 ± 1.11) and the MVC + ESWT group (0.75 ± 0.47).

A subgroup analysis of identical reconstructive approaches revealed no statistically significant differences.

When the number of lymphatic vessels within different nerve segments of each group were compared, no statistically significant differences were found either.

## 4. Discussion

This study’s main hypothesis was that regeneration of the murine median nerve following reconstruction with ANGs, i.e., the gold-standard method, or with MVCs, i.e., nonneural tissue, can be enhanced by a single postoperative application of low-energy defocused ESWT. The pro-regenerative effects of ESWT have been shown in the context of various musculoskeletal and neurological diseases, including carpal tunnel syndrome [50,51,52,53], spinal cord injury [54,55,56,57,58] and PNI in vivo [17,19,20,27,58,59,60]. Of note, to the best of our knowledge, all the PNI studies were performed in the sciatic nerve model of the rat, and four of the seven studies we retrieved featured a sciatic crush injury [19,58,59,60], e.g., axonotmesis. In regard to the sciatic nerve injury model, it must be noted that this model has some significant disadvantages, the most noteworthy being the development of neuropathic pain and automutilations in case of neurotmesis injuries and difficulties assessing functional recovery by means of walking track or gait analysis [28]. Considering the severity of the nerve injury, it must be noted that while crush-type nerve injuries in humans are likely to recover ad integrum even in the absence of any surgical or pharmacological treatment, this occurs even faster in rodents given the profound differences in neurobiology and speed of axonal regeneration between the species [61]. Neurotmesis injuries, however, are likely to show incomplete or even insufficient functional recovery even in case of optimal surgical treatment [47]. We, therefore, reasoned it to be essential to investigate the effects of ESWT to enhance functional recovery following neurotmesis of the rat median nerve. Given that the use of nerve autografts to reconstruct peripheral nerves is limited by their restricted availability and the resulting donor site morbidity, we also deemed it indispensable to evaluate whether positive effects of ESWT can also be observed following median nerve reconstruction with nonneuronal tissue, e.g., with MVCs.

First of all, the promising reports of studies evaluating functional recovery following nerve reconstruction with MVCs in humans [43,44,62] could not be reproduced in our study. This is in line with the findings of others who showed that functional recovery following nerve reconstruction by means of MVCs was markedly inferior in comparison to autologous nerve grafting in rodent models [38,63]. In one of our previous works, we suggested that this might be related to a higher number of proteases in the murine genome, resulting in increased protein turnover and, therefore, accelerated degeneration of muscle fibers within the MVC, which, in turn, might hamper axonal regeneration due to a decrease in the MVC’s intraluminal diameter [45]. This was also supported by the intraoperative findings we observed at WPO12 in regard to the diameter of the distal segments of MVCs.

More importantly, despite some trends for a favorable functional outcome in the group of animals receiving ESWT in addition to nerve reconstruction with an ANG, our study’s results do not support the positive reports of other preclinical works regarding the neuroregenerative effects of ESWT both on peripheral nerves [17,19,27,59,64] and the spinal cord [55,56,58]. The results of the grasping test did not verify a significant positive effect of ESWT on functional recovery following reconstruction of the median nerve with either ANGs or MVCs. Interpretation of this test’s results was further hampered by the high number of animals which had to be excluded from statistical analysis due to limited motivation to participate in the assessment, a problematic observation which has also been reported by other authors and might relate to unpleasant sensations experienced by animals when held by the tail [38,46].

The electrophysiological evaluations at the end of our observation period were not indicative of any significant direct pro-regenerative effect of a single ESWT application following median nerve reconstruction. Interestingly, these measurements revealed a significantly lower FDS CMAP amplitude area in the rats which underwent nerve repair with an MVC + ESWT as compared to the ANG-treated animals which also received postoperative ESWT. As no other significant differences were observable between the groups, this indicates either a slightly pro-regenerative effect of ESWT following autologous nerve grafting, a disadvantageous effect of ESWT in case of nerve repair with MVCs or both. The wet muscle weight of the FDS revealed a similar trend and also emphasized the superiority of nerve repair with ANGs as compared to MVCs as reported by us and other authors [38,45,63,65].

Computerized gait analysis with the CatWalk device showed a trend for better functional recovery in the rats which underwent nerve repair with ANGs in comparison to MVCs, too. Interestingly, analysis of paw print dimensions, i.e., print area, print length and print width hinted towards improved recovery in the rats of the ANG + ESWT group, especially in regard to the print area ratio. Additionally, the ANG + ESWT-treated animals had a significantly higher paw width ratio than those which received an MVC, supporting our theory that ESWT exerts positive effects on nerve regeneration and functional recovery after autograft repair. The observed increase in the swing time and the stand index ratio following segmental median nerve injury and reconstruction is in line with what we described previously and emphasizes the value of these two parameters, especially of the stand index ratio, to assess functional deficits following median nerve neurotmesis [39]. Interestingly, our subgroup analysis of identical reconstructive approaches showed a significant difference in the front paw (FP) base of support between the ANG group and the ANG + ESWT group in WPO2. As most parameters of gait, changes in the BoS can be induced by several different factors, e.g., an increase in the BoS can account for an unstable gait following central nervous lesions [66,67,68], and a decreased BoS was reported after sciatic nerve neurotmesis in rats [69], whereas it remained not significantly changed following neurotmesis of the rat femoral nerve [33] and the median nerve [39]. This might, on the one hand, be related to the relatively high functional deficit following sciatic nerve neurotmesis in contrast to median and femoral nerve neurotmesis [33,39]. In this context, an early work involving use of the CatWalk device in a rat model of sciatic nerve injury postulated that the observed in the BoS is mainly related to a separate motor dysfunction rather that an adaptive response to other functional losses [70]. However, one could also assume that a significant loss of innervated, i.e., sensate plantar paw surface following peripheral nerve injury (PNI) is likely counterbalanced by placing the affected paw and its contralateral counterpart closer together to account for this functional loss. Additionally, the experience of mechanical allodynia during ambulation might also influence the BoS, whereas in our study, one would expect that the symptoms at WPO2 were most likely caused by collateral sprouting of intact adjacent peripheral nerves in the paw, e.g., the ulnar nerve into the original territory of the median nerve [71]. In our opinion, one should also consider the experience of local pain at the operation site as a likely mechanism, and a study published in 2018 reported an increase in the FP BoS following nerve reconstruction with a conventional nerve flap as opposed to a decrease in the FP BoS following median nerve excision, autograft repair and noteworthy sham surgery [72]. As the exact reasons for the significant difference in the FP BoS in WPO2 remain to be elucidated in detail, we postulate that this could be related to the direct positive effects of ESWT on wound healing on the site of operation [73]. Additionally, it was reported that ESWT induces selective loss of unmyelinated, i.e., nociceptive nerve fibers with potential analgesic effects due to selective denervation of target organs. [74,75,76].

Regarding quantification of axons within the reconstructed median nerve at WPO12, two main findings require discussion. First of all, we observed a significant decrease in axon numbers in MVCs when comparing the proximal nerve sections with the distal ones. This finding is in total accordance with our and others’ observation that axonal regeneration through MVCs is inferior in comparison to that through ANGs, most likely because axonal regrowth is hindered in case the muscle fibers within the MVC are degraded before the regrowing axons reach the distal segment of the MVC [38,45,63] as observable by the narrowing of the distal MVC segments in our study. In addition, extensive formation of coaptation neuroma at the proximal repair site was reported by the beforementioned authors and was also observable at WPO12 in our study. It has been emphasized that in order to achieve the best possible result when performing nerve reconstruction with an MVC, it is essential to pull the nerve stumps into the MVC rather than just coaptate them to the proximal and distal stumps [43,44]. However, this is more difficult and technically challenging in a rat model due to the small diameter of the harvested veins used to fabricate the MVC [45]. Secondly, we observed a significantly smaller number of axons in the proximal and distal nerve segments of the MVC + ESWT-treated animals in comparison to the MVC group. This finding also points towards disadvantageous, i.e., regeneration-hindering effects of ESWT, in the context of nerve repairs with MVCs.

The role of vascularization and neoangiogenesis and their respective assessment has gained increasing attention recently [72,77,78,79,80,81,82,83,84,85,86,87,88]. In our study, significantly lower numbers of blood vessels were found in the distal nerve segments of the MVCs which were treated with ESWT when compared to the rats of the MVC group. In accordance with the previous paragraph, these findings provide evidence that the observed effects of ESWT on peripheral nerve regeneration in our study, especially in case of the MVCs, were at least partially related to hindered neovascularization of the regenerating nerve. As ESWT was shown to exert proangiogenic effects in several other studies [21,22,57,64,89,90,91], the underlying reasons for the observations in our study remain to be elucidated.

Our study was also among the first [49,77,92,93] to shine light on the involvement of lymphatic vasculature and lymphangiogenesis, respectively, in peripheral nerve repair and regeneration. Interestingly, we found that the number of lymphatics was drastically increased in both ANGs and MCVs following median nerve reconstruction. These vessels were especially prominent in the middle portions of the reconstructed nerve segments. Saffari et al. have recently shown that hemangiogenesis in peripheral nerve regeneration occurs from both stumps of the original nerve but primarily from the proximal one [86]. In our study, we observed a relatively higher number of lymphatic vessels in the middle graft segments of both MVCs and ANGs. We hypothesize that this observation can be explained by a study recently published by our group. In this work, we showed for the first time that Schwann cells induce apoptosis of lymphatic endothelial cells when cocultured in vitro via extended filopodia-like protrusions [49]. While this, on the one hand, explains the absence of lymphatic vessels inside uninjured murine peripheral nerves, these findings can be further extrapolated to the findings in the study at hand. As Schwann cells were shown to induce apoptosis of lymphatic endothelial cells in vitro, we hypothesized that the reduced numbers of lymphatic vessels in the proximal and distal segments of ANGs are related to the presence of Schwann cells which migrate into the reconstructed nerve from both the proximal stump and the distal nerve stump [94], inducing apoptosis of lymphatic vessels in these areas. The higher number of lymphatic vessels in MVCs can be explained by the fact that they contain fewer Schwann cells in addition to pro-angiogenetic effects exerted by the vein component of MVCs [95,96]. Although it has been recently hypothesized [92] and later shown [93] that lymphangiogenesis plays an important role in peripheral nerve regeneration following axonotmesis, our results also indicate that higher numbers of lymphatic vasculature do not necessarily lead to better functional recovery, as illustrated by the MVC + ESWT group in our study. Given the complexity of the peripheral nervous system and the intricate interplay of its components, it is reasonable to assume that “the more the better” does not necessarily apply in this context. Although not statistically significant, we observed a trend towards lower numbers of lymphatic vessels in the distal nerve segments of the ESWT-treated ANGs as compared to the ANG group, which is in accordance with what we observed regarding the number of blood vessels in the reconstructed median nerves. As aforementioned, ESWT was shown to exert major pro-angiogenetic effects in vivo [22,91], making interpretation of these results more difficult. However, Hausner et al. showed that ESWT did not increase the number of blood vessels in a sciatic nerve autografting model [27]. Circling back to the potential interplay of Schwann cells and lymphatic vasculature in vivo, we hypothesize that the lower number of lymphatic vessels might be explained by direct effects of ESWT on Schwann cells which, in turn, indirectly affect lymphatic endothelial cells. As it was shown that the ex vivo ESWT-treated Schwann cells showed increased proliferative activity and—upon respective inductive cues—expression of myelin-associated phenotypic markers [25], they might exert negative effects on lymphatic endothelial vasculature cells in vivo. Given the few published reports [17,24,25,97] addressing the effects of ESWT on Schwann cells, we advise for further studies to elucidate the underlying reasons for this phenomenon.

Our study bears several limitations. First of all, the majority of the animals had to be excluded from statistical analysis of the grasping test. This was mainly because the animals showed fluctuating and reduced motivation to participate in repeated measurements over the course of the postoperative observation period. This problem was reported by other authors in the past [38] and is especially concerning when Lewis rats are used in this test, which is still considered the gold-standard method for the evaluation of functional recovery in rat models of median nerve injury [36]. Given the reduced number of animals eligible for further statistical comparison of grasping strength and grasping ability, results of this comparison should be interpreted with utmost caution. Secondly, while our study pioneers a standardized application of ESWT to promote peripheral nerve repair via a 3D-printed holder, the ideal application intensity and frequency of ESWT remain to be elucidated in further studies. This might have resulted in significantly higher energies on the target tissue than during a conventional, free-hand application of ESWT and subsequent negative effects on axonal regeneration. As higher-energy ESWT is more effective in regard to the achieved treatment effects, so are the side effects, e.g., the aforementioned destruction of unmyelinated nerve fibers [98] and very high intensities are almost certainly more harmful than regeneration-promoting [22].

## 5. Conclusions

Our study investigated a novel application method for ESWT in axonal regeneration as well as the effects of ESWT on the formation of blood and lymphatic vessels in the regenerating nerve after autologous nerve grafting or muscle-in-vein conduit repair. A single postoperative application of defocused low-intensity extracorporeal shockwave therapy did not significantly enhance neuroregeneration in a rat model of segmental median nerve injury but decreased the number of blood and lymphatic vessels within the regenerated nerves. The rats treated with MVCs showed a worse functional recovery than those treated with an ANG.

## Figures and Tables

**Table 1:** Summary of functional recovery as assessed by the grasping ability (1/3: no toe flexion; 2/3: toe flexion without measurable strength; 3/3: toe flexion with measurable strength) during the 12-week postoperative observation period. Statistical differences were tested with the nonparametric Kruskal–Wallis test and the Dunn–Bonferroni post hoc test. ANG: autologous nerve graft (*n* = 3); MVC: muscle-in-vein conduit (*n* = 5), ANG + ESWT: autologous nerve graft with postoperative extracorporeal shockwave therapy (*n* = 6), MVC + ESWT: muscle-in-vein conduit with postoperative extracorporeal shockwave therapy (*n* = 7), n. s.: not significant, WPO: postoperative week.

**WPO1**							
Ability	1/3	2/3	3/3	vs. ANG	vs. MVC	vs. ANG + ESWT	vs. MVC + ESWT
ANG	3/3 (100%)	0/3 (0%)	0/3 (0%)		n. s.	n. s.	n. s.
MVC	5/5 (100%)	0/5 (0%)	0/5 (0%)	n. s.		n. s.	n. s.
ANG + ESWT	6/6 (100%)	0/6 (0%)	0/6 (0%)	n. s.	n. s.		n. s.
MVC + ESWT	7/7 (100%)	0/7 (0%)	0/7 (0%)	n. s.	n. s.	n. s.	
**WPO2**							
Ability	1/3	2/3	3/3	vs. ANG	vs. MVC	vs. ANG + ESWT	vs. MVC + ESWT
ANG	3/3 (100%)	0/3 (0%)	0/3 (0%)		n. s.	n. s.	n. s.
MVC	5/5 (100%)	0/5 (0%)	0/5 (0%)	n. s.		n. s.	n. s.
ANG + ESWT	6/6 (100%)	0/6 (0%)	0/6 (0%)	n. s.	n. s.		n. s.
MVC + ESWT	7/7 (100%)	0/7 (0%)	0/7 (0%)	n. s.	n. s.	n. s.	
**WPO3**							
Ability	1/3	2/3	3/3	vs. ANG	vs. MVC	vs. ANG + ESWT	vs. MVC + ESWT
ANG	3/3 (100%)	0/3 (0%)	0/3 (0%)		n. s.	n. s.	n. s.
MVC	5/5 (100%)	0/5 (0%)	0/5 (0%)	n. s.		n. s.	n. s.
ANG + ESWT	5/6 (83.3%)	1/6 (16.7%)	0/6 (0%)	n. s.	n. s.		n. s.
MVC + ESWT	7/7 (100%)	0/7 (0%)	0/7 (0%)	n. s.	n. s.	n. s.	
**WPO4**							
Ability	1/3	2/3	3/3	vs. ANG	vs. MVC	vs. ANG + ESWT	vs. MVC + ESWT
ANG	0/3 (0%)	2/3 (66%)	1/3 (33%)		***p*** ** < 0.05**	n. s.	***p*** ** < 0.05**
MVC	5/5 (100%)	0/5 (0%)	0/5 (0%)	***p*** ** < 0.05**		n. s.	n. s.
ANG + ESWT	3/6 (50%)	2/6 (33.3%)	1/6 (16.7%)	n. s.	n. s.		n. s.
MVC + ESWT	7/7 (100%)	0/7 (0%)	0/7 (0%)	***p*** ** < 0.05**	n. s.	n. s.	
**WPO5**							
Ability	1/3	2/3	3/3	vs. ANG	vs. MVC	vs. ANG + ESWT	vs. MVC + ESWT
ANG	0/3 (0%)	1/3 (33.3%)	2/3 (66.7%)		***p*** ** < 0.05**	n. s.	***p*** ** < 0.05**
MVC	5/5 (100%)	0/5 (0%)	0/5 (0%)	***p*** ** < 0.05**		n. s.	n. s.
ANG + ESWT	2/6 (33.3%)	2/6 (33.3%)	2/6 (33.3%)	n. s.	n. s.		n. s.
MVC + ESWT	6/7 (85.7%)	1/7 (14.3%)	0/7 (0%)	n. s.	n. s.	***p*** **< 0.05**	
**WPO6**							
Ability	1/3	2/3	3/3	vs. ANG	vs. MVC	vs. ANG + ESWT	vs. MVC + ESWT
ANG	0/3 (0%)	0/3 (0%)	3/3 (100%)		***p*** ** < 0.05**	n. s.	***p*** ** < 0.05**
MVC	5/5 (100%)	0/5 (0%)	0/5 (0%)	***p*** ** < 0.05**		***p*** ** < 0.05**	n. s.
ANG + ESWT	0/6 (0%)	2/6 (33.3%)	4/6 (66.7%)	n. s.	***p*** **< 0.05**		***p*** ** < 0.05**
MVC + ESWT	6/7 (85.7%)	1/7 (14.3%)	0/7 (0%)	***p*** ** < 0.05**	n. s.	***p*** ** < 0.05**	
**WPO7**							
Ability	1/3	2/3	3/3	vs. ANG	vs. MVC	vs. ANG + ESWT	vs. MVC + ESWT
ANG	0/3 (0%)	0/3 (0%)	3/3 (100%)		***p*** ** < 0.05**	n. s.	n. s.
MVC	5/5 (100%)	0/5 (0%)	0/5 (0%)	***p*** ** < 0.05**		***p*** ** < 0.05**	n. s.
ANG + ESWT	0/6 (0%)	2/6 (33.3%)	4/6 (66.7%)	n. s.	***p*** ** < 0.05**		n. s.
MVC + ESWT	5/7 (71.4%)	1/7 (14.3%)	1/7 (14.3%)	n. s.	n. s.	n. s.	
**WPO8**							
Ability	1/3	2/3	3/3	vs. ANG	vs. MVC	vs. ANG + ESWT	vs. MVC + ESWT
ANG	0/3 (0%)	0/3 (0%)	3/3 (100%)		n. s.	n. s.	n. s.
MVC	4/5 (80%)	1/5 (20%)	0/5 (0%)	n. s.		***p*** ** < 0.05**	n. s.
ANG + ESWT	0/6 (0%)	1/6 (16.7%)	5/6 (83.3%)	n. s.	***p*** ** < 0.05**		n. s.
MVC + ESWT	4/7 (57.1%)	1/7 (14.3%)	2/7 (28.6%)	n. s.	n. s.	n. s.	
**WPO9**							
Ability	1/3	2/3	3/3	vs. ANG	vs. MVC	vs. ANG + ESWT	vs. MVC + ESWT
ANG	0/3 (0%)	0/3 (0%)	3/3 (100%)		n. s.	n. s.	n. s.
MVC	4/5 (80%)	1/5 (20%)	0/5 (0%)	n. s.		***p*** ** < 0.05**	n. s.
ANG + ESWT	0/6 (0%)	0/6 (0%)	6/6 (100%)	n. s.	***p*** ** < 0.05**		n. s.
MVC + ESWT	4/7 (57.1%)	0/7 (0%)	33/7 (42.9%)	n. s.	n. s.	n. s.	
**WPO10**							
Ability	1/3	2/3	3/3	vs. ANG	vs. MVC	vs. ANG + ESWT	vs. MVC + ESWT
ANG	0/3 (0%)	0/3 (0%)	3/3 (100%)		n. s.	n. s.	n. s.
MVC	3/5 (60%)	1/5 (20%)	1/5 (20%)	n. s.		n. s.	n. s.
ANG + ESWT	0/6 (0%)	0/6 (0%)	6/6 (100%)	n. s.	n. s.		n. s.
MVC + ESWT	4/7 (57.1%)	0/7 (0%)	33/7 (42.9%)	n. s.	n. s.	n. s.	
**WPO11**							
Ability	1/3	2/3	3/3	vs. ANG	vs. MVC	vs. ANG + ESWT	vs. MVC + ESWT
ANG	0/3 (0%)	0/3 (0%)	3/3 (100%)		n. s.	n. s.	n. s.
MVC	3/5 (60%)	0/5 (20%)	2/5 (40%)	n. s.		n. s.	n. s.
ANG + ESWT	0/6 (0%)	0/6 (0%)	6/6 (100%)	n. s.	n. s.		n. s.
MVC + ESWT	3/7 (42.9%)	1/7 (14.3%)	3/7 (42.9%)	n. s.	n. s.	n. s.	
**WPO12**							
Ability	1/3	2/3	3/3	vs. ANG	vs. MVC	vs. ANG + ESWT	vs. MVC + ESWT
ANG	0/3 (0%)	0/3 (0%)	3/3 (100%)		n. s.	n. s.	n. s.
MVC	3/5 (60%)	0/5 (20%)	2/5 (40%)	n. s.		n. s.	n. s.
ANG + ESWT	0/6 (0%)	0/6 (0%)	6/6 (100%)	n. s.	n. s.		n. s.
MVC + ESWT	1/7 (14.3%)	3/7 (42.9%)	3/7 (42.9%)	n. s.	n. s.	n. s.	

## Data Availability

The datasets generated for this study are available from the corresponding author on reasonable request.

## Supplementary Materials

The following supporting information can be downloaded at: https://www.mdpi.com/article/10.3390/biomedicines10081777/s1, Figure S1: Representative schematics and finished design of the device for ESWT application; Figure S2: Example qualitative output of the deep neural network model on the validation dataset, Figure S3: Representative photomicrographs of anti-CD31 stained consecutive cross-sections through the regenerated tissue in the proximal nerve segment, middle of the nerve graft and distal nerve graft. Table S1: Raw data of the deep neural network model on the validation dataset on axon quantification.

## References

[1] Huckhagel T., Nuchtern J., Regelsberger J., Lefering R., TraumaRegister DGU (2018). Nerve injury in severe trauma with upper extremity involvement: Evaluation of 49,382 patients from the TraumaRegister DGU(R) between 2002 and 2015. Scand. J. Trauma Resusc. Emerg. Med..

[2] Huckhagel T., Nüchtern J., Regelsberger J., Gelderblom M., Lefering R., TraumaRegister DGU (2018). Nerve trauma of the lower extremity: Evaluation of 60,422 leg injured patients from the TraumaRegister DGU^®^ between 2002 and 2015. Scand. J. Trauma Resusc. Emerg. Med..

[3] Karsy M., Watkins R., Jensen M.R., Guan J., Brock A.A., Mahan M.A. (2019). Trends and Cost Analysis of Upper Extremity Nerve Injury Using the National (Nationwide) Inpatient Sample. World Neurosurg..

[4] Foster C.H., Karsy M., Jensen M.R., Guan J., Eli I., Mahan M.A. (2019). Trends and Cost-Analysis of Lower Extremity Nerve Injury Using the National Inpatient Sample. Neurosurgery.

[5] Heinzel J.C., Dadun L.F., Prahm C., Winter N., Bressler M., Lauer H., Ritter J., Daigeler A., Kolbenschlag J. (2021). Beyond the Knife—Reviewing the Interplay of Psychosocial Factors and Peripheral Nerve Lesions. J. Pers. Med..

[6] Bergmeister K.D., Grosse-Hartlage L., Daeschler S.C., Rhodius P., Bocker A., Beyersdorff M., Kern A.O., Kneser U., Harhaus L. (2020). Acute and long-term costs of 268 peripheral nerve injuries in the upper extremity. PLoS ONE.

[7] Seddon H.J. (1943). Three Types of Nerve Injury. Brain.

[8] Sunderland S. (1951). A classification of peripheral nerve injuries producing loss of function. Brain.

[9] Pan D., Mackinnon S.E., Wood M.D. (2020). Advances in the repair of segmental nerve injuries and trends in reconstruction. Muscle Nerve.

[10] Bassilios Habre S., Bond G., Jing X.L., Kostopoulos E., Wallace R.D., Konofaos P. (2018). The Surgical Management of Nerve Gaps: Present and Future. Ann. Plast. Surg..

[11] Kornfeld T., Vogt P.M., Radtke C. (2019). Nerve grafting for peripheral nerve injuries with extended defect sizes. Wien. Med. Wochenschr..

[12] Fowler J.R., Lavasani M., Huard J., Goitz R.J. (2015). Biologic strategies to improve nerve regeneration after peripheral nerve repair. J. Reconstr. Microsurg..

[13] Carvalho C.R., Oliveira J.M., Reis R.L. (2019). Modern Trends for Peripheral Nerve Repair and Regeneration: Beyond the Hollow Nerve Guidance Conduit. Front. Bioeng. Biotechnol..

[14] Navarro X. (2016). Functional evaluation of peripheral nerve regeneration and target reinnervation in animal models: A critical overview. Eur. J. Neurosci..

[15] Hussain G., Wang J., Rasul A., Anwar H., Qasim M., Zafar S., Aziz N., Razzaq A., Hussain R., de Aguilar J.L.G. (2020). Current status of therapeutic approaches against peripheral nerve injuries: A detailed story from injury to recovery. Int. J. Biol. Sci..

[16] Martínez de Albornoz P., Delgado P.J., Forriol F., Maffulli N. (2011). Non-surgical therapies for peripheral nerve injury. Br. Med. Bull..

[17] Li H.X., Zhang Z.C., Peng J. (2021). Low-intensity extracorporeal shock wave therapy promotes recovery of sciatic nerve injury and the role of mechanical sensitive YAP/TAZ signaling pathway for nerve regeneration. Chin. Med. J..

[18] Peng D., Tan Y., Reed-Maldonado A.B., Lin G., Lue T.F. (2020). Molecular mechanism of action of low-intensity extracorporeal shockwave therapy for regenerating penile and peripheral nerves. Turk. J. Urol..

[19] Park H.J., Hong J., Piao Y., Shin H.J., Lee S.J., Rhyu I.J., Yi M.H., Kim J., Kim D.W., Beom J. (2020). Extracorporeal shockwave therapy enhances peripheral nerve remyelination and gait function in a crush model. Adv. Clin. Exp. Med..

[20] Sağir D., Bereket C., Onger M.E., Bakhit N., Keskin M., Ozkan E. (2019). Efficacy of Extracorporeal Shockwaves Therapy on Peripheral Nerve Regeneration. J. Craniofac. Surg..

[21] Simplicio C.L., Purita J., Murrell W., Santos G.S., Dos Santos R.G., Lana J. (2020). Extracorporeal shock wave therapy mechanisms in musculoskeletal regenerative medicine. J. Clin. Orthop. Trauma.

[22] Hausner T., Nogradi A. (2013). The use of shock waves in peripheral nerve regeneration: New perspectives?. Int. Rev. Neurobiol..

[23] Murata R., Ohtori S., Ochiai N., Takahashi N., Saisu T., Moriya H., Takahashi K., Wada Y. (2006). Extracorporeal shockwaves induce the expression of ATF3 and GAP-43 in rat dorsal root ganglion neurons. Auton. Neurosci..

[24] Hercher D., Redl H., Schuh C. (2020). Motor and sensory Schwann cell phenotype commitment is diminished by extracorporeal shockwave treatment in vitro. J. Peripher. Nerv. Syst..

[25] Schuh C.M., Hausner T., Redl H.R. (2016). A therapeutic shock propels Schwann cells to proliferate in peripheral nerve injury. Brain Circ..

[26] Wang B., Ning H., Reed-Maldonado A.B., Zhou J., Ruan Y., Zhou T., Wang H.S., Oh B.S., Banie L., Lin G. (2017). Low-Intensity Extracorporeal Shock Wave Therapy Enhances Brain-Derived Neurotrophic Factor Expression through PERK/ATF4 Signaling Pathway. Int. J. Mol. Sci..

[27] Hausner T., Pajer K., Halat G., Hopf R., Schmidhammer R., Redl H., Nogradi A. (2012). Improved rate of peripheral nerve regeneration induced by extracorporeal shock wave treatment in the rat. Exp. Neurol..

[28] Irintchev A. (2011). Potentials and limitations of peripheral nerve injury models in rodents with particular reference to the femoral nerve. Ann. Anat..

[29] Haastert-Talini K., Phillips J., Hercher D., Hausner T. (2020). Appropriate Animal Models for Translational Nerve Research. Peripheral Nerve Tissue Engineering and Regeneration.

[30] Haastert-Talini K., Haastert-Talini K., Assmus H., Antoniadis G. (2017). Peripheral Nerve Tissue Engineering: An Outlook on Experimental Concepts. Modern Concepts of Peripheral Nerve Repair.

[31] de Ruiter G.C., Spinner R.J., Verhaagen J., Malessy M.J. (2014). Misdirection and guidance of regenerating axons after experimental nerve injury and repair. J. Neurosurg..

[32] Dellon A.L., Mackinnon S.E. (1989). Sciatic nerve regeneration in the rat. Validity of walking track assessment in the presence of chronic contractures. Microsurgery.

[33] Heinzel J.C., Hercher D., Redl H. (2020). The course of recovery of locomotor function over a 10-week observation period in a rat model of femoral nerve resection and autograft repair. Brain Behav..

[34] Bertelli J.A., Taleb M., Saadi A., Mira J.C., Pecot-Dechavassine M. (1995). The rat brachial plexus and its terminal branches: An experimental model for the study of peripheral nerve regeneration. Microsurgery.

[35] Bertelli J.A., Orsal D., Mira J.C. (1994). Median nerve neurotization by peripheral nerve grafts directly implanted into the spinal cord: Anatomical, behavioural and electrophysiological evidences of sensorimotor recovery. Brain Res..

[36] Ronchi G., Morano M., Fregnan F., Pugliese P., Crosio A., Tos P., Geuna S., Haastert-Talini K., Gambarotta G. (2019). The Median Nerve Injury Model in Pre-clinical Research—A Critical Review on Benefits and Limitations. Front. Cell. Neurosci..

[37] Papalia I., Tos P., Stagno d’Alcontres F., Battiston B., Geuna S. (2003). On the use of the grasping test in the rat median nerve model: A re-appraisal of its efficacy for quantitative assessment of motor function recovery. J. Neurosci. Methods.

[38] Stößel M., Rehra L., Haastert-Talini K. (2017). Reflex-based grasping, skilled forelimb reaching, and electrodiagnostic evaluation for comprehensive analysis of functional recovery-The 7-mm rat median nerve gap repair model revisited. Brain Behav..

[39] Heinzel J.C., Oberhauser V., Keibl C., Swiadek N., Längle G., Frick H., Kolbenschlag J., Prahm C., Grillari J., Hercher D. (2021). Evaluation of Functional Recovery in Rats After Median Nerve Resection and Autograft Repair Using Computerized Gait Analysis. Front. Neurosci..

[40] Daeschler S.C., Harhaus L., Bergmeister K.D., Boecker A., Hoener B., Kneser U., Schoenle P. (2018). Clinically Available Low Intensity Ultrasound. Devices do not Promote Axonal Regeneration After Peripheral Nerve Surgery-A Preclinical Investigation of an FDA-Approved Device. Front. Neurol..

[41] Casal D., Mota-Silva E., Iria I., Pais D., Farinho A., Alves S., Pen C., Mascarenhas-Lemos L., Ferreira-Silva J., Ferraz-Oliveira M. (2020). Functional and Physiological Methods of Evaluating Median Nerve Regeneration in the Rat. JoVE.

[42] Ahmad I., Mir M.A., Khan A.H. (2017). An Evaluation of Different Bridging Techniques for Short Nerve Gaps. Ann. Plast. Surg..

[43] Manoli T., Schulz L., Stahl S., Jaminet P., Schaller H.E. (2014). Evaluation of sensory recovery after reconstruction of digital nerves of the hand using muscle-in-vein conduits in comparison to nerve suture or nerve autografting. Microsurgery.

[44] Ederer I.A., Mayer J.A., Heinzel J., Kolbenschlag J., Daigeler A., Wahler T. (2022). Outcome After Reconstruction of 43 Digital Nerve Defects With Muscle-in-Vein Conduits. J. Hand Surg..

[45] Heinzel J.C., Quyen Nguyen M., Kefalianakis L., Prahm C., Daigeler A., Hercher D., Kolbenschlag J. (2021). A systematic review and meta-analysis of studies comparing muscle-in-vein conduits with autologous nerve grafts for nerve reconstruction. Sci. Rep..

[46] Bertelli J.A., Mira J.C. (1995). The grasping test: A simple behavioral method for objective quantitative assessment of peripheral nerve regeneration in the rat. J. Neurosci. Methods.

[47] Heinzel J., Langle G., Oberhauser V., Hausner T., Kolbenschlag J., Prahm C., Grillari J., Hercher D. (2020). Use of the CatWalk gait analysis system to assess functional recovery in rodent models of peripheral nerve injury—A systematic review. J. Neurosci. Methods.

[48] Heinzel J., Swiadek N., Ashmwe M., Ruhrnossl A., Oberhauser V., Kolbenschlag J., Hercher D. (2020). Automated Gait Analysis to Assess Functional Recovery in Rodents with Peripheral Nerve or Spinal Cord Contusion Injury. J. Vis Exp..

[49] Hromada C., Hartmann J., Oesterreicher J., Stoiber A., Daerr A., Schädl B., Priglinger E., Teuschl-Woller A.H., Holnthoner W., Heinzel J. (2022). Occurrence of Lymphangiogenesis in Peripheral Nerve Autografts Contrasts Schwann Cell-Induced Apoptosis of Lymphatic Endothelial Cells In Vitro. Biomolecules.

[50] Turgut M.C., Saglam G., Toy S. (2021). Efficacy of extracorporeal shock wave therapy for pillar pain after open carpal tunnel release: A double-blind, randomized, sham-controlled study. Korean J. Pain.

[51] Li W., Dong C., Wei H., Xiong Z., Zhang L., Zhou J., Wang Y., Song J., Tan M. (2020). Extracorporeal shock wave therapy versus local corticosteroid injection for the treatment of carpal tunnel syndrome: A meta-analysis. J. Orthop. Surg. Res..

[52] Chang C.Y., Chen L.C., Chou Y.C., Li T.Y., Ho T.Y., Wu Y.T. (2020). The Effectiveness of Platelet-Rich Plasma and Radial Extracorporeal Shock Wave Compared with Platelet-Rich Plasma in the Treatment of Moderate Carpal Tunnel Syndrome. Pain Med..

[53] Xu D., Ma W., Jiang W., Hu X., Jiang F., Mao C., Wang Y., Fang L., Luo N., Li H. (2020). A randomized controlled trial: Comparing extracorporeal shock wave therapy versus local corticosteroid injection for the treatment of carpal tunnel syndrome. Int. Orthop..

[54] Leister I., Mittermayr R., Mattiassich G., Aigner L., Haider T., Machegger L., Kindermann H., Grazer-Horacek A., Holfeld J., Schaden W. (2022). The effect of extracorporeal shock wave therapy in acute traumatic spinal cord injury on motor and sensory function within 6 months post-injury: A study protocol for a two-arm three-stage adaptive, prospective, multi-center, randomized, blinded, placebo-controlled clinical trial. Trials.

[55] Matsuda M., Kanno H., Sugaya T., Yamaya S., Yahata K., Handa K., Shindo T., Shimokawa H., Ozawa H., Itoi E. (2020). Low-energy extracorporeal shock wave therapy promotes BDNF expression and improves functional recovery after spinal cord injury in rats. Exp. Neurol..

[56] Wang L., Jiang Y., Jiang Z., Han L. (2016). Effect of low-energy extracorporeal shock wave on vascular regeneration after spinal cord injury and the recovery of motor function. Neuropsychiatr. Dis. Treat..

[57] Yahata K., Kanno H., Ozawa H., Yamaya S., Tateda S., Ito K., Shimokawa H., Itoi E. (2016). Low-energy extracorporeal shock wave therapy for promotion of vascular endothelial growth factor expression and angiogenesis and improvement of locomotor and sensory functions after spinal cord injury. J. Neurosurg. Spine.

[58] Lee J.H., Kim S.G. (2015). Effects of extracorporeal shock wave therapy on functional recovery and neurotrophin-3 expression in the spinal cord after crushed sciatic nerve injury in rats. Ultrasound. Med. Biol..

[59] Seo M., Lim D., Kim S., Kim T., Kwon B.S., Nam K. (2021). Effect of Botulinum Toxin Injection and Extracorporeal Shock Wave Therapy on Nerve Regeneration in Rats with Experimentally Induced Sciatic Nerve Injury. Toxins.

[60] Lee J.H., Cho S.H. (2013). Effect of extracorporeal shock wave therapy on denervation atrophy and function caused by sciatic nerve injury. J. Phys. Sci..

[61] Kaplan H.M., Mishra P., Kohn J. (2015). The overwhelming use of rat models in nerve regeneration research may compromise designs of nerve guidance conduits for humans. J. Mater. Sci. Mater. Med..

[62] Schiefer J.L., Schulz L., Rath R., Stahl S., Schaller H.E., Manoli T. (2015). Comparison of short- with long-term regeneration results after digital nerve reconstruction with muscle-in-vein conduits. Neural Regen. Res..

[63] Stößel M., Wildhagen V.M., Helmecke O., Metzen J., Pfund C.B., Freier T., Haastert-Talini K. (2018). Comparative Evaluation of Chitosan Nerve Guides with Regular or Increased Bendability for Acute and Delayed Peripheral Nerve Repair: A Comprehensive Comparison with Autologous Nerve Grafts and Muscle-in-Vein Grafts. Anat. Rec..

[64] Yamaya S., Ozawa H., Kanno H., Kishimoto K.N., Sekiguchi A., Tateda S., Yahata K., Ito K., Shimokawa H., Itoi E. (2014). Low-energy extracorporeal shock wave therapy promotes vascular endothelial growth factor expression and improves locomotor recovery after spinal cord injury. J. Neurosurg..

[65] Geuna S., Tos P., Raimondo S., Lee J.M., Gambarotta G., Nicolino S., Fornaro M., Papalia I., Perroteau I., Battiston B. (2007). Functional, morphological and biomolecular assessment of posttraumatic neuro-muscular recovery in the rat forelimb model. Acta Neurochir. Suppl..

[66] Hamers F.P., Lankhorst A.J., van Laar T.J., Veldhuis W.B., Gispen W.H. (2001). Automated quantitative gait analysis during overground locomotion in the rat: Its application to spinal cord contusion and transection injuries. J. Neurotrauma.

[67] Joosten E.A., Veldhuis W.B., Hamers F.P. (2004). Collagen containing neonatal astrocytes stimulates regrowth of injured fibers and promotes modest locomotor recovery after spinal cord injury. J. Neurosci. Res..

[68] Datto J.P., Shah A.K., Bastidas J.C., Arheart K.L., Marcillo A.E., Dietrich W.D., Pearse D.D. (2016). Use of the CatWalk Gait Device to Assess Differences in Locomotion between Genders in Rats Inherently and following Spinal Cord Injury. Dataset Pap. Sci..

[69] Deumens R., Jaken R.J., Marcus M.A., Joosten E.A. (2007). The CatWalk gait analysis in assessment of both dynamic and static gait changes after adult rat sciatic nerve resection. J. Neurosci. Methods.

[70] Bozkurt A., Deumens R., Scheffel J., O'Dey D.M., Weis J., Joosten E.A., Fuhrmann T., Brook G.A., Pallua N. (2008). CatWalk gait analysis in assessment of functional recovery after sciatic nerve injury. J. Neurosci. Methods.

[71] Cobianchi S., de Cruz J., Navarro X. (2014). Assessment of sensory thresholds and nociceptive fiber growth after sciatic nerve injury reveals the differential contribution of collateral reinnervation and nerve regeneration to neuropathic pain. Exp. Neurol..

[72] Casal D., Mota-Silva E., Iria I., Alves S., Farinho A., Pen C., Lourenco-Silva N., Mascarenhas-Lemos L., Silva-Ferreira J., Ferraz-Oliveira M. (2018). Reconstruction of a 10-mm-long median nerve gap in an ischemic environment using autologous conduits with different patterns of blood supply: A comparative study in the rat. PLoS ONE.

[73] Mittermayr R., Antonic V., Hartinger J., Kaufmann H., Redl H., Teot L., Stojadinovic A., Schaden W. (2012). Extracorporeal shock wave therapy (ESWT) for wound healing: Technology, mechanisms, and clinical efficacy. Wound Repair Regen..

[74] Hausdorf J., Lemmens M.A., Heck K.D., Grolms N., Korr H., Kertschanska S., Steinbusch H.W., Schmitz C., Maier M. (2008). Selective loss of unmyelinated nerve fibers after extracorporeal shockwave application to the musculoskeletal system. Neuroscience.

[75] Arcilla C.K., Tadi P. (2022). Neuroanatomy, Unmyelinated Nerve Fibers. StatPearls.

[76] Ohtori S., Inoue G., Mannoji C., Saisu T., Takahashi K., Mitsuhashi S., Wada Y., Takahashi K., Yamagata M., Moriya H. (2001). Shock wave application to rat skin induces degeneration and reinnervation of sensory nerve fibres. Neurosci. Lett..

[77] Zucal I., Mihic-Probst D., Pignet A.L., Calcagni M., Giovanoli P., Frueh F.S. (2022). Intraneural fibrosis and loss of microvascular architecture—Key findings investigating failed human nerve allografts. Ann. Anat..

[78] Saffari S., Saffari T.M., Ulrich D.J.O., Hovius S.E.R., Shin A.Y. (2021). The interaction of stem cells and vascularity in peripheral nerve regeneration. Neural Regen. Res..

[79] Masgutov R., Zeinalova A., Bogov A., Masgutova G., Salafutdinov I., Garanina E., Syromiatnikova V., Idrisova K., Mullakhmetova A., Andreeva D. (2021). Angiogenesis and nerve regeneration induced by local administration of plasmid pBud-coVEGF165-coFGF2 into the intact rat sciatic nerve. Neural Regen. Res..

[80] Prahm C., Heinzel J., Kolbenschlag J., Phillips J., Hercher D., Hausner T. (2021). Blood Supply and Microcirculation of the Peripheral Nerve. Peripheral Nerve Tissue Engineering and Regeneration.

[81] Saffari T.M., Mathot F., Friedrich P.F., Bishop A.T., Shin A.Y. (2021). Surgical Angiogenesis of Decellularized Nerve Allografts Improves Early Functional Recovery in a Rat Sciatic Nerve Defect Model. Plast. Reconstr. Surg..

[82] Saffari T.M., Mathot F., Thaler R., van Wijnen A.J., Bishop A.T., Shin A.Y. (2021). Microcomputed analysis of nerve angioarchitecture after combined stem cell delivery and surgical angiogenesis to nerve allograft. J. Plast. Reconstr. Aesthetic Surg..

[83] Saffari T.M., Badreldin A., Mathot F., Bagheri L., Bishop A.T., van Wijnen A.J., Shin A.Y. (2020). Surgical angiogenesis modifies the cellular environment of processed nerve allografts in a rat sciatic nerve defect model. Gene.

[84] Saffari T.M., Mathot F., Bishop A.T., Shin A.Y. (2020). New methods for objective angiogenesis evaluation of rat nerves using microcomputed tomography scanning and conventional photography. Microsurgery.

[85] Saffari T.M., Mathot F., Friedrich P.F., Bishop A.T., Shin A.Y. (2020). Revascularization patterns of nerve allografts in a rat sciatic nerve defect model. J. Plast. Reconstr. Aesthetic Surg..

[86] Saffari T.M., Bedar M., Hundepool C.A., Bishop A.T., Shin A.Y. (2020). The role of vascularization in nerve regeneration of nerve graft. Neural Regen. Res..

[87] Mathot F., Rbia N., Bishop A.T., Hovius S.E.R., Shin A.Y. (2020). Adipose derived mesenchymal stem cells seeded onto a decellularized nerve allograft enhances angiogenesis in a rat sciatic nerve defect model. Microsurgery.

[88] Muangsanit P., Shipley R.J., Phillips J.B. (2018). Vascularization Strategies for Peripheral Nerve Tissue Engineering. Anat. Rec..

[89] Harwood A.E., Green J., Cayton T., Raza A., Wallace T., Carradice D., Chetter I.C., Smith G.E. (2018). A feasibility double-blind randomized placebo-controlled trial of extracorporeal shockwave therapy as a novel treatment for intermittent claudication. J. Vasc. Surg..

[90] Cayton T., Harwood A., Smith G.E., Chetter I. (2016). A Systematic Review of Extracorporeal Shockwave Therapy as a Novel Treatment for Intermittent Claudication. Ann. Vasc. Surg..

[91] Leu S., Huang T.H., Chen Y.L., Yip H.K. (2017). Effect of Extracorporeal Shockwave on Angiogenesis and Anti-Inflammation: Molecular-Cellular Signaling Pathways. Transl. Res. Biomed..

[92] Frueh F.S., Gousopoulos E., Power D.M., Ampofo E., Giovanoli P., Calcagni M., Laschke M.W. (2020). A potential role of lymphangiogenesis for peripheral nerve injury and regeneration. Med. Hypotheses.

[93] Meng F.W., Jing X.N., Song G.H., Jie L.L., Shen F.F. (2020). Prox1 induces new lymphatic vessel formation and promotes nerve reconstruction in a mouse model of sciatic nerve crush injury. J. Anat..

[94] Chen B., Chen Q., Parkinson D.B., Dun X.-P. (2019). Analysis of Schwann Cell Migration and Axon Regeneration Following Nerve Injury in the Sciatic Nerve Bridge. Front. Mol. Neurosci..

[95] Saio S., Konishi K., Hohjoh H., Tamura Y., Masutani T., Iddamalgoda A., Ichihashi M., Hasegawa H., Mizutani K.I. (2021). Extracellular Environment-Controlled Angiogenesis, and Potential Application for Peripheral Nerve Regeneration. Int. J. Mol. Sci..

[96] Geuna S., Tos P., Battiston B., Giacobini-Robecchi M.G. (2004). Bridging peripheral nerve defects with muscle-vein combined guides. Neurol. Res..

[97] Schuh C.M., Hercher D., Stainer M., Hopf R., Teuschl A.H., Schmidhammer R., Redl H. (2016). Extracorporeal shockwave treatment: A novel tool to improve Schwann cell isolation and culture. Cytotherapy.

[98] Park K.D., Lee W.Y., Park M.H., Ahn J.K., Park Y. (2018). High- versus low-energy extracorporeal shock-wave therapy for myofascial pain syndrome of upper trapezius: A prospective randomized single blinded pilot study. Medicine.

