# Supplementary figures and images for: ESWT Diminishes Axonal Regeneration following Repair of the Rat Median Nerve with Muscle-In-Vein Conduits but Not after Autologous Nerve Grafting
Source: Biomedicines. 2022 Jul 22;10(8):1777. doi: 10.3390/biomedicines10081777 (PMC9394363; doi:10.3390/biomedicines10081777)

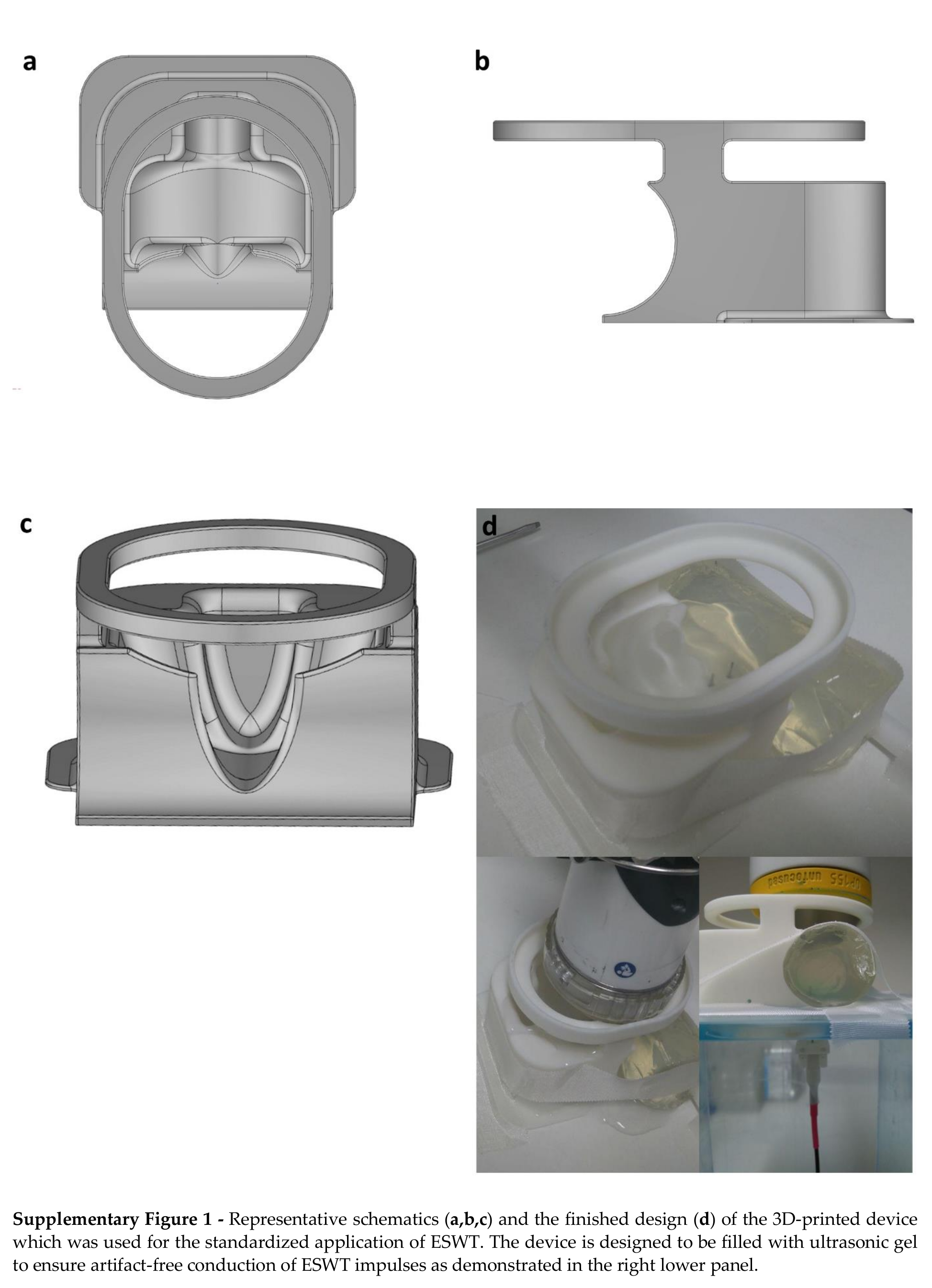

Supplement: Supplementary file 1 [file biomedicines-10-01777-s001.zip › Supplementary Figure S1.tiff]

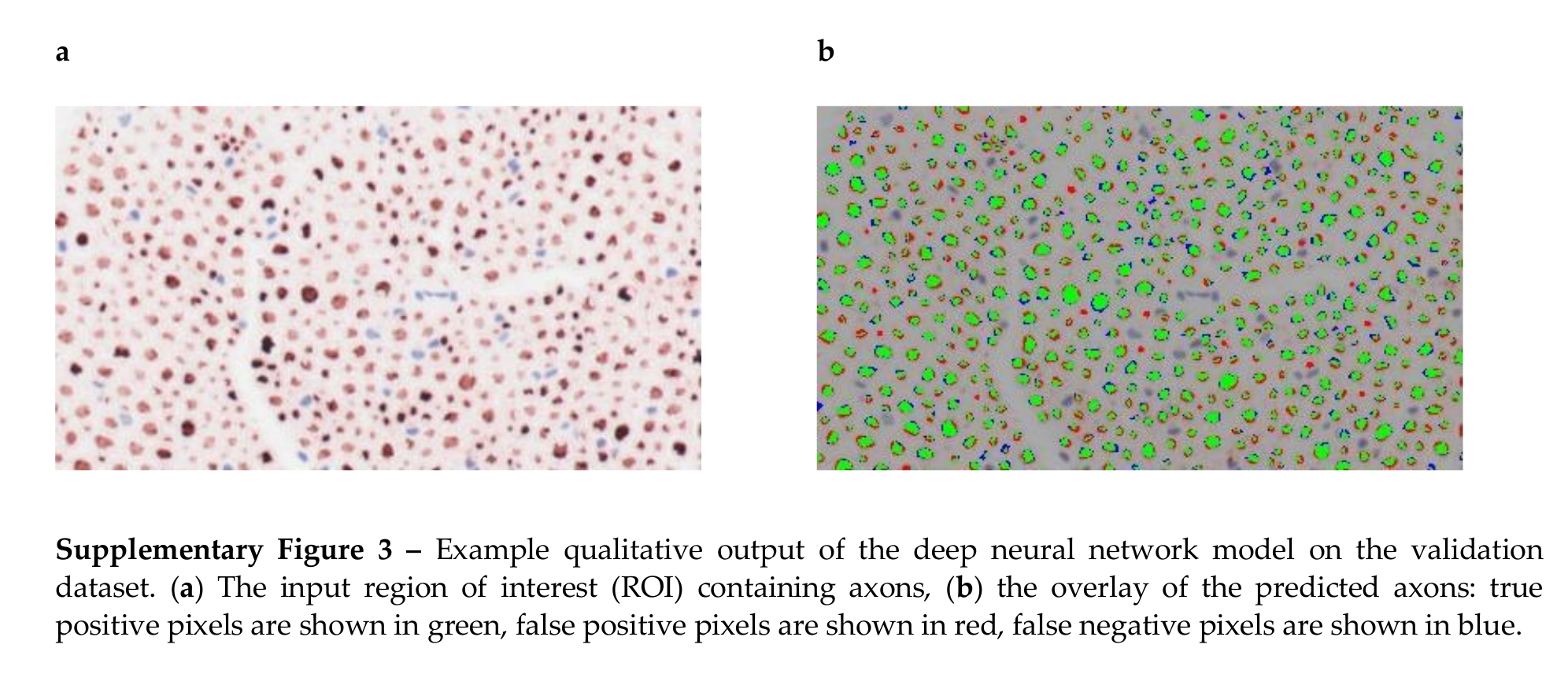

Supplement: Supplementary file 1 [file biomedicines-10-01777-s001.zip › Supplementary Figure S2.tiff]

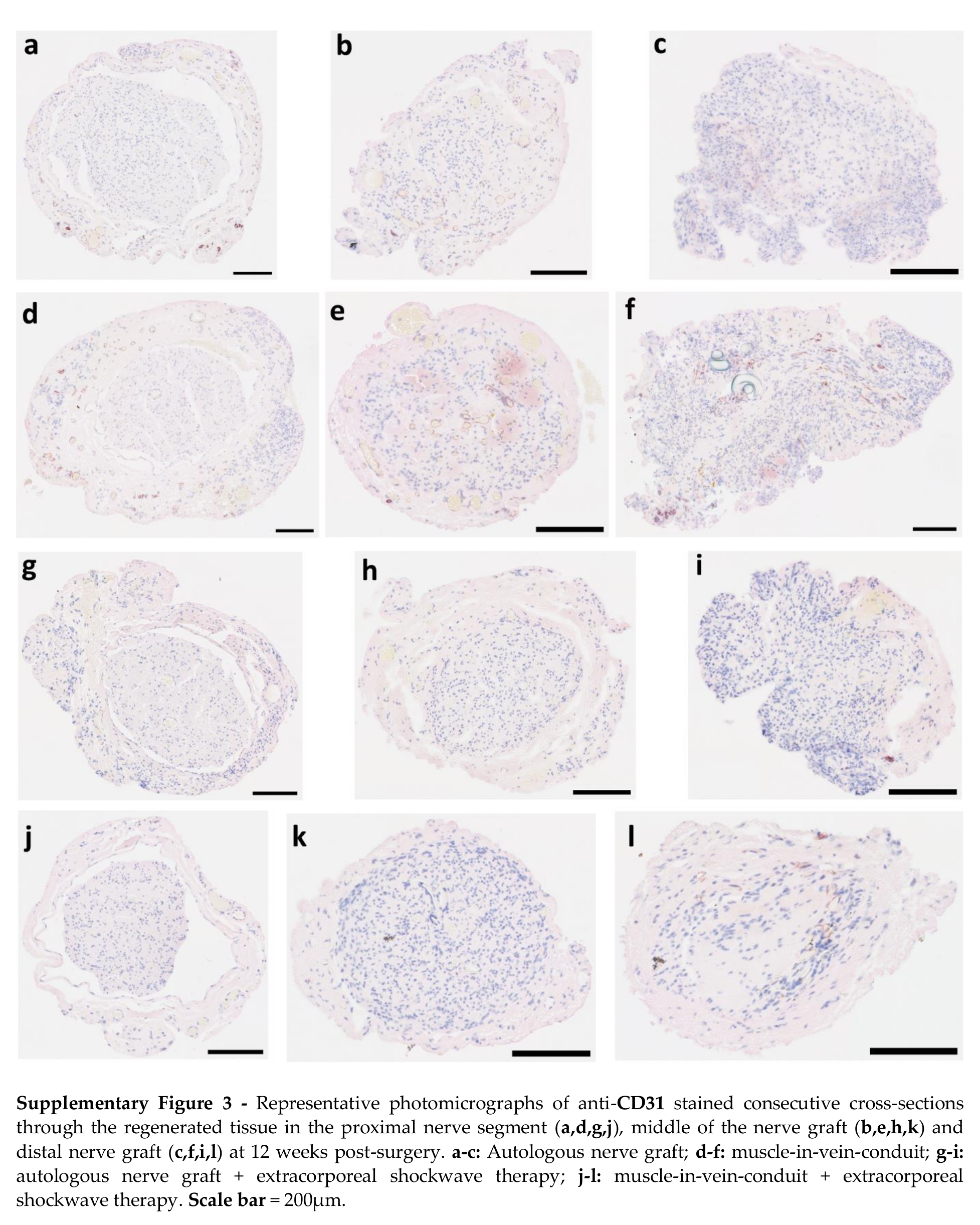

Supplement: Supplementary file 1 [file biomedicines-10-01777-s001.zip › Supplementary Figure S3.tiff]
